# The Effects of Exercise and Activity-Based Physical Therapy on Bone after Spinal Cord Injury

**DOI:** 10.3390/ijms23020608

**Published:** 2022-01-06

**Authors:** Tommy W. Sutor, Jayachandra Kura, Alex J. Mattingly, Dana M. Otzel, Joshua F. Yarrow

**Affiliations:** 1 Research Service, Malcom Randall Department of Veterans Affairs Medical Center, North Florida/South Georgia Veterans Health System, Gainesville, FL 32608, USA; Thomas.Sutor@va.gov (T.W.S.); jayachankura@ufl.edu (J.K.); 2 Brain Rehabilitation Research Center, Malcom Randall Department of Veterans Affairs Medical Center, North Florida/South Georgia Veterans Health System, Gainesville, FL 32608, USA; dotzel@ufl.edu; 3 Geriatrics Research, Education, and Clinical Center, North Florida/South Georgia Veterans Health System, Gainesville, FL 32608, USA; mattingly@ufl.edu; 4 Division of Endocrinology, Diabetes, and Metabolism, University of Florida College of Medicine, Gainesville, FL 32611, USA

**Keywords:** neuromuscular electrical stimulation, bodyweight supported treadmill training, vibration, osteoblast, osteoclast, osteocyte, sclerostin, Wnt beta catenin, RANKL, OPG

## Abstract

Spinal cord injury (SCI) produces paralysis and a unique form of neurogenic disuse osteoporosis that dramatically increases fracture risk at the distal femur and proximal tibia. This bone loss is driven by heightened bone resorption and near-absent bone formation during the acute post-SCI recovery phase and by a more traditional high-turnover osteopenia that emerges more chronically, which is likely influenced by the continual neural impairment and musculoskeletal unloading. These observations have stimulated interest in specialized exercise or activity-based physical therapy (ABPT) modalities (e.g., neuromuscular or functional electrical stimulation cycling, rowing, or resistance training, as well as other standing, walking, or partial weight-bearing interventions) that reload the paralyzed limbs and promote muscle recovery and use-dependent neuroplasticity. However, only sparse and relatively inconsistent evidence supports the ability of these physical rehabilitation regimens to influence bone metabolism or to increase bone mineral density (BMD) at the most fracture-prone sites in persons with severe SCI. This review discusses the pathophysiology and cellular/molecular mechanisms that influence bone loss after SCI, describes studies evaluating bone turnover and BMD responses to ABPTs during acute versus chronic SCI, identifies factors that may impact the bone responses to ABPT, and provides recommendations to optimize ABPTs for bone recovery.

## 1. Introduction

An estimated 250,000 to 500,000 new spinal cord injuries (SCI) occur worldwide each year [1], with males representing ~80% of the population [2]. Roughly one-third of these are motor-complete SCIs that result in permanent sublesional paralysis, while the remainder are incomplete and retain voluntary contractility in some muscles that are innervated below the lesion [2]. Locomotor dysfunction is the most recognizable symptom of SCI and is accompanied by other medical consequences that develop in this population [3], including severe osteoporosis and high fracture risk [4], which worsen with increasing SCI severity [5,6,7,8,9] and injury duration [10,11].

Bone loss after SCI is termed neurogenic or disuse osteoporosis and is confined to the sublesional skeleton [10,11,12], with the most rapid and prevalent bone deficits occurring at the distal femur and proximal tibia regions [11,13,14]. At these sites, 50–100% lower trabecular bone mineral density (BMD) develops in individuals within the first two to three years of SCI [11,13,14], and 40–80% lower cortical bone mass exists several years after injury [14]. Some evidence also indicates that cortical bone becomes more porous after SCI [15]. Collectively, these bone deficits imply a significant weakening of skeletal integrity which likely influences the 20- to 100-fold higher fracture risk in persons with SCI when compared with the general population [16].

After SCI, fractures are usually non-traumatic, due to the mobility limitations in persons with SCI, and result from low-velocity compressive forces or torsional stresses [17] that develop while seated or during transfers to, or falls from, a wheelchair. These fractures most commonly occur at the epiphysis or metaphysis [5] of the distal femur [18] or proximal tibia [7,8], where bone loss is the most severe and may require extended inpatient hospitalization [5]. Moreover, a single fracture more than doubles the risk for other secondary medical comorbidities after SCI, including venous thromboembolic events, respiratory illnesses, and pressure ulcers, among others [19]. These comorbidities influence the 30% higher five-year mortality risk for those of any age who fracture after SCI and the more than three-fold higher five-year mortality risk for persons with SCI who fracture after age 50 years [20]. The severe bone loss, high fracture incidence, and the associated morbidity and mortality indicate the need to improve osteoporosis screening and to develop evidence-based guidelines to prevent and treat osteoporosis in the SCI population [21,22].

## 2. Determining BMD and Fracture Risk after SCI

Dual-energy X-ray absorptiometry (DXA) is the standard to assess osteoporosis and fracture risk at traditional osteoporosis sites (e.g., lumbar spine and hip), via the quantification of areal (a)BMD and T-scores [23]. Specialized DXA techniques have also been developed to assess distal femur and/or proximal tibia aBMD after SCI [17,24,25], although not all the DXA systems are capable of imaging these locations and T-scores have not been established at these sites. As such, some have suggested using traditional osteoporosis sites as surrogates for the distal femur and proximal tibia. However, BMD changes occur more rapidly at the knee than at other bone sites after SCI [26]. Furthermore, after SCI, aBMD changes at the knee are only moderately correlated with the total hip and femoral neck aBMD and T-scores [26,27] and may not correspond with the degree of bone loss at the hip or femoral neck [28,29,30,31], with significant predictive inaccuracy between the sites that surround the knee and hip [27]. Alternatively, peripheral quantitative computerized tomography (pQCT) yields volumetric (v)BMD of the trabecular and cortical bone compartments and has been used to estimate vBMD fracture thresholds at the distal femur epiphysis (<114 mg/cm^3^) and distal tibia epiphysis (<71 mg/cm^3^) in persons with SCI [32]. High-resolution (HR)-pQCT with finite element analysis (FEA) [33] can also measure vBMD, along with the bone microstructural parameters, and can simultaneously model bone tensile properties, providing insight into bone microarchitecture and mechanical alterations that contribute to increased fracture risk after SCI [34,35]. For example, the reduction in proximal femur bone strength that was estimated via FEA was three times greater than the aBMD loss that was determined by DXA over the first few months after SCI [36], likely because DXA cannot discern trabecular vs. cortical BMD nor quantify other bone parameters that influence fracture risk [37]. Regardless, the limited availability of pQCT systems restricts their practicality and highlights the continued need for DXA evaluations to identify fracture risk in persons with SCI.

## 3. Pathophysiology of SCI-Induced Bone Loss

Within the SCI population, persons with complete paralysis display the most extensive bone loss [9,26] and highest fracture risk [8], likely because the residual voluntary muscle function lessens bone loss. As evidence, persons with incomplete SCI display less bone loss in the lesser impaired limb [38]. Moreover, cast immobilization (a technique that limits muscle contraction and restricts voluntary joint motions) has been shown to worsen bone loss in a rat severe SCI model [39], demonstrating that even a minimal degree of residual muscle contractility that accompanies severe SCI assists in preserving BMD. These observations support the understanding that disuse is a factor mediating SCI-induced bone loss. However, bone loss after SCI is distinct from that which is occurring in response to other disuse conditions in both severity and mechanism. For example, in humans with complete SCI, trabecular and cortical bone loss occurs at a rate nearing 1% per week over the initial few months post-SCI [40], which is 4–10 times faster than in other types of disuse (e.g., prolonged bed rest or microgravity exposure) [41]. Similarly, in rodent SCI models, bone loss is two or more times faster than that resulting from cast immobilization [39] or sciatic neurectomy [42]. These findings suggest that other factors that are occurring secondary to SCI may worsen bone loss, including systemic hormonal changes, altered bone innervation [43], and/or impaired bone perfusion [44]. For further discussion, readers are directed to the following review [4].

## 4. Bone Turnover after SCI

Bone undergoes continuous remodeling throughout the lifespan, which is balanced during homeostasis via integrated resorption and formation processes that maintain skeletal integrity. However, after severe SCI, a unique form of unopposed bone resorption drives bone loss [4]. As evidence, Minaire et al. examined persons with SCI and observed signs of increased osteoclastic resorption that accompanied a near-absence of surface-level bone formation at the iliac crest [45] that is suggestive of uncoupled bone turnover. Circulating bone resorption markers are also several-fold higher than the upper reference ranges in persons with SCI throughout the acute (<four-months) to subacute (4–12 months) post-injury periods when bone loss is most rapid, while circulating bone formation markers remain near reference ranges [10,46,47,48,49,50,51]. Similarly, in rodent models of severe SCI, the use of dynamic histomorphometry has revealed that trabecular bone resorption persists at the distal femur and proximal tibia in the near absence of bone formation throughout the initial one to three-weeks post-SCI [44,52,53,54,55,56,57], when nearly all the trabecular bone loss occurs. Thereafter, trabecular bone formation renormalizes [58,59] and bone loss slows [52].

## 5. Mechanisms Regulating Bone Loss after SCI

The molecular mechanisms that propagate the uncoupled bone turnover that is present in the paralyzed limbs after SCI and that drive the exceedingly rapid bone loss in this population, in comparison with other disuse conditions, require further elucidation. Given that disuse is a factor that mediates SCI-induced bone loss, it is likely that osteocytes (primary bone mechanosensor) influence neurogenic osteoporosis. Osteocytes reside within the calcified bone matrix and communicate with other osteocytes and with osteoclasts, osteoblasts, and other cells that reside on bone surfaces via dendritic projections that emerge from the osteocyte cell body to form an interconnected dendritic network. Dendrites provide one means by which the osteocytes sense alterations in localized bone strains that result from disuse or imposed loading and transduce this information to the osteocyte cell body, a process that is referred to as mechanotransduction. In response to this stimulus, osteocytes release a host of nuclear-derived signaling molecules (e.g., receptor activator of NF-κB ligand (RANKL), osteoprotegrin (OPG), sclerostin, and others) that orchestrate osteoclastic and/or osteoblastic bone remodeling. Readers are directed to the following reviews that discuss osteocyte mechanosensors and mechanotransduction-associated signaling pathways [60,61].

While it is likely that osteocytes orchestrate the skeletal responses to SCI, few studies have directly assessed this possibility or described how osteocytes respond to paralysis or to imposed bone loading after SCI. Qin et al. observed osteocyte morphological aberrations in rats within seven-weeks of spinal transition, including reductions in the dendritic length and dendritic number, along with altered osteocyte cell body shape [62]. The molecular mechanisms that regulate these morphologic changes require further elucidation. However, administration of a monoclonal sclerostin antibody that binds and inactivates the circulating sclerostin was shown to preserve the dendritic length and osteocyte cell body morphology in this model [62], suggesting potential autocrine regulation. Regardless, it remains unknown whether the osteocyte morphological aberrations that are occurring in the paralyzed limbs produce functional consequences (e.g., impaired ability to detect imposed bone strains) within the dendritic network.

## 6. RANKL Signaling

RANKL is an osteocyte-derived protein that is necessary for the differentiation of hematopoietic progenitors of the monocyte-macrophage lineage into osteoclasts. RANKL stimulates osteoclastogenesis and bone resorption by binding RANK receptors on the cell surfaces of osteoclast precursors and osteoclasts, respectively [63]. RANKL-mediated osteoclastic resorption is primarily modified in response to secreted concentrations of RANKL and OPG, an endogenous decoy receptor for RANKL that is produced by osteoblast-lineage cells and prevents RANK binding. The relative abundance of RANKL to OPG is a key factor that influences RANKL signaling, with higher RANKL and/or lower OPG stimulating bone resorption and osteoclastogenesis. Readers are directed to the following review for an overview of RANKL signaling in bone biology [63].

Signs of altered RANKL signaling coexist with bone loss in rodent SCI models. For example, cultured bone marrow mesenchymal or stromal cells that were isolated from spinalized mice exhibit higher RANKL and lower OPG vs. the controls, which may underlie the two- to three-fold increase in tartrate-resistant acid phosphatase (TRAP)+ osteoclast-like cells that develop in bone marrow culture that are derived from spinalized mice [64]. Similarly, in rodent SCI models, RANKL mRNA and protein expressions were 75–300% higher and OPG mRNA and protein were 30–75% lower at the distal femur and proximal tibia when compared with the controls. Recently, Cirnigliaro et al. [65] reported that 12-mo treatment with Denosumab, a human monoclonal antibody that binds and antagonizes RANKL activity, completely prevented distal femur and proximal tibia aBMD loss in a small cohort of persons with acute SCI. Gifre et al. reported that Denosumab increased total hip, femoral neck, and lumbar spine aBMD in persons with subacute to chronic SCI [66], with BMD gains being associated with the magnitude of RANKL suppression [67]. Collectively, these data indicate that rodent SCI models display an altered RANKL:OPG ratio in a manner that would be expected to promote bone loss, and that pharmacologic RANKL inhibition prevents aBMD loss at the most fracture prone sites after SCI and increases aBMD at other sites.

Recently, RANK [68] and RANKL [69] have also been identified as functional components of extracellular vesicles (EVs) that are secreted directly from osteoclasts and osteoblasts, respectively, providing a mechanism for intercellular communication [70]. In this regard, RANK in osteoclast-derived EVs can bind osteoblastic RANKL and stimulate a reverse RANKL signaling pathway that promotes bone formation and that couples bone resorption and formation processes [71]. Osteoblast-derived RANKL-containing EVs can also promote osteoclastogenesis in vivo, at least when delivered to transgenic RANKL deficient mice [72]. Given these observations, it is enticing to imagine that the uncoupled bone turnover that is present in the paralyzed limbs after SCI may be influenced by transient alterations in RANK or RANKL containing EVs, although, this remains to be determined.

## 7. Canonical Wnt/β-Catenin Signaling and Sclerostin

The canonical Wnt/β-catenin signaling cascade promotes bone formation by stimulating osteoblast differentiation and osteoblast growth rate, by inhibiting osteoblast apoptosis, and by stimulating osteoblast activity. This signaling pathway is initiated when the various Wnt ligands (e.g., Wnt1 and Wnt3a) bind the low-density lipoprotein receptor-related protein (LRP)5/6 and Frizzled receptor complex on osteoblasts and prevent the Axin-adenomatous polyposis coli-glycogen synthase kinase 3-casein kinase 1 (APC-GSK-3-Ck1) complex from phosphorylating cytoplasmic β-catenin, which traditionally marks β-catenin for proteasomal degradation. In response, unphosphorylated β-catenin accumulates within the cytoplasm, where it can be shuttled to the nucleus to interact with DNA binding proteins (e.g., TCF/LEF) to promote the activation of Wnt responsive gene transcription pathways [61]. Several negative regulators of Wnt-signaling exist, including sclerostin, which binds the LRP5/6 receptors and prevents Wnt ligands from initiating Wnt-signaling. Sclerostin is known to mediate bone loss that develops in response to unloading [73] and bone gain resulting from imposed skeletal strains, with sclerostin suppression being essential for the osteogenic responses to mechanical loading [74]. Readers are directed to the following review for further discussion on Wnt signaling in bone [61].

Alterations in Wnt signaling accompany bone loss in the paralyzed limbs in rodent SCI models. For example, several proteins that are involved in Wnt signaling (LRP5, Wnt1, and Wnt3) are repressed at the distal femur and proximal tibia within a few weeks of SCI, while the number of osteocytes that stain for sclerostin are increased [75] and SOST (gene encoding sclerostin) is 300% higher. These changes likely influence the 50% lower β-catenin protein expression that has been observed at the proximal tibia after SCI, along with the reduced β-catenin expression that is present in cultured mesenchymal cells that are derived from spinal transected mice. Elevated sclerostin also appears to mediate SCI-induced bone loss, given that SOST-/- mice are protected against bone loss and suppressed osteoblastogenesis after spinal transition [76]. Moreover, a pharmacologic sclerostin antibody that binds and inactivates sclerostin has been shown to completely prevent distal femur and proximal tibia bone loss in rodents when it is delivered immediately after severe incomplete [53] to complete SCI [62] by promoting bone formation, and to reverse trabecular and cortical bone loss when it is delivered after bone loss emerges [77].

## 8. Activity-Based Physical Therapy (ABPT) after SCI

Given the role of disuse in mediating the musculoskeletal decline after SCI, a large body of research has focused on ABPTs and other exercise-based regimens that reload the impaired limbs to restore muscle integrity [78]. Common ABPTs include overground locomotor training and/or bodyweight-supported treadmill training (BWSTT) that is accompanied by manual or robotic-assisted placement of the impaired limbs into normal gait patterns, passive cycling, or functional electrical stimulation (FES) that is coupled with cycling, rowing, or resistance training (RT). With intense repetitive training, ABPTs are theorized to activate and optimize sublesional spinal networks, enabling the improved performance of task-specific motor activities [79]. For example, BWSTT and other locomotor-based modalities have shown promise in promoting use-dependent neuroplasticity after mild to moderate motor-incomplete SCI and result in improved walking speed, temporal gait parameters, and lower limb muscle activation patterns [80,81,82], along with increased muscle strength and rate of torque development in some persons with incomplete SCI [78]. Structural and functional plasticity likely drives motor recovery resulting from these ABPTs and stems from the reorganization of both supraspinal and spinal cord neural circuits [83,84]. As evidence, beneficial adaptations to spinal neuronal pathways have been observed in response to ABPT by probing soleus Hoffmann reflex during walking in which homosynaptic facilitation normalized, homosynaptic depression reversed, and presynaptic inhibition of Ia afferents improved [83,85,86]. The functional recovery in persons with incomplete SCI undergoing ABPT has also been associated with findings that indicate greater descending corticospinal drive, including increased ankle dorsiflexor and knee extensor maximal motor-evoked potentials, a probe of corticospinal tract excitability [87], and improved ankle dorsiflexor and plantar flexor muscle co-activation patterns during walking [88]. Possible mechanisms underlying ABPT-mediated neuroplasticity may involve the upregulation of brain-derived neurotrophic factor and/or its receptor, tyrosine kinase B mRNA, in the spinal cord, which mediate improvements of synaptic transmission, axon regeneration, and motor neuron survival [89]. For further discussion on this topic, we refer readers to the following review [78].

## 9. Effects of ABPT and Reloading Modalities on Bone after SCI

In uninjured persons, weight-bearing exercise that produces high peak strains and/or high strain rates increases BMD [90] and prevents disuse-mediated bone loss [91]. In various models, cyclical loading has also been shown to stimulate bone formation and increase bone mass in a manner that is dependent upon the compressive strain that is applied to bone, the loading frequency (Hz), and the loading duration. In contrast, stationary/static loading does not typically alter bone parameters [92]. This knowledge has contributed to an emphasis on ABPTs to increase BMD after SCI [17] and to recommendations from healthcare providers to utilize weight-bearing activities to improve BMD after SCI [93]. However, evidence demonstrating the skeletal benefits of ABPTs is sparse in persons with SCI with several meta-analyses concluding that insufficient evidence exists to establish their effectiveness in improving BMD [94,95]. Likewise, the existing evidence is contradictory when assessing the ability of ABPTs to alter bone turnover in a manner that would be expected to improve BMD after SCI. For example, Bloomfield et al. [96] reported that nine-months of FES cycling increased serum osteocalcin (bone formation marker) >75% in persons with chronic complete SCI. Similarly, Mobarke et al. [97] reported that 12-weeks of BWSTT starting at 50% bodyweight support and progressing to full weight-bearing increased osteocalcin when compared with stretching and RT exercises, while others have reported that six- to eight-months of BWSTT suppressed urinary deoxypyridinoline/creatinine (bone resorption marker) without altering osteocalcin [50]. In comparison, Craven et al. [98] reported that those with chronic incomplete SCI displayed no change in circulating osteocalcin, CTX (bone resorption marker), or sclerostin in response to four-months of FES assisted BWSTT. Similarly, others reported that persons with incomplete to complete SCI exhibit little change in circulating bone formation markers or circulating/urinary bone resorption markers in response to FES-based cycling [30] or RT [51,99], BWSTT [100], seated [101] or standing vibration [102], or a multimodal ABPT regimen [103].

Relatively few preclinical SCI studies have evaluated the possibility that ABPTs alter bone turnover or improve BMD, likely because few preclinical modalities exist to reload the paralyzed limbs. In this regard, Qin et al. developed an implantable electrical stimulation (ES) system that elicited unilateral near-maximal contractions of the paralyzed soleus and plantaris muscles in spinal transected rats, with ES delivered 60 min/day for one-week (40 Hz at 1.5 V, (2 s on/18 s off), 1.5 V) [64]. This brief ES protocol improved plantaris muscle mass but did not alter BMD or bone microstructure when compared with SCI rats not receiving ES. However, ES suppressed circulating CTX by ~50% and reduced the number of osteoclast-like TRAP-positive osteoclasts that develop in ex vivo cultures, suggesting ES suppressed osteoclastogenesis and bone resorption. ES also reversed the suppression of OPG mRNA in ex vivo osteoblast cultures, without altering RANKL mRNA, and suppressed mRNA expression of SOST, along with several other genes that negatively regulate Wnt-signaling. Despite these changes, ES did not reverse the SCI-induced suppression of osteocalcin nor did it alter the reduction in alkaline phosphatase-positive or von Kossa-stained colonies that develop in ex vivo osteoblast cultures that are derived from tibia or femur bone marrow, suggesting that one-week of ES did not stimulate osteoblastogenesis or bone formation. More recently, the same group reported that a four-week ES protocol lessened distal femur and proximal tibia aBMD loss and increased several trabecular and cortical bone microstructural variables at the distal femur and femoral midshaft [104]. This four-week ES protocol also increased the circulating osteocalcin, suggesting an increased whole-body bone formation. However, distal femur mineralizing surface (index of active bone formation), mineral apposition rate (index of osteoblast activity), and surface-level bone formation were similarly suppressed in SCI+ES and SCI groups vs. the controls, providing no direct evidence that ES stimulated bone formation at this site. Furthermore, ES did not prevent the suppression of osteoblastogenesis that resulted from SCI, nor did it increase osteoblast SOST mRNA expression. However, ES reduced the RANKL:OPG mRNA ratio within the hindlimbs, reduced the number of TRAP+ osteoclasts in ex vivo cultures, and produced a 50% reduction in the percentage of trabecular bone that was covered by osteoclasts when compared with the untreated SCI animals, providing evidence of an antiresorptive effect.

Beyond the studies that are described above, we are aware of only a few others assessing bone responses to loading in preclinical SCI models. Zamarioli et al. [105] evaluated two loading protocols in spinal transected rats: (1) ES of the quadriceps and triceps surae muscles [300 µs pulses, 50 Hz (5 s on/15 s off), 20–150 mA, 3 days/week, 30 min/day] and (2) a custom-built bipedal standing frame that resulted in 70% of total body mass being supported by the hindlimbs. Neither modality prevented distal femur or proximal tibia aBMD loss nor lessened the reduction in bone strength at these sites, although, the maximal strength of the lumbar vertebrae increased by ~30% in response to bipedal standing. We have also reported that hindlimb cast immobilization worsened trabecular and cortical bone loss at the distal femur and proximal tibia and reduced the distal femur bone strength in a severe SCI model [39]. In this study, two-weeks of quadrupedal (q)BWSTT reversed the bone loss that resulted from cast immobilization but did not lessen bone loss that resulted directly from SCI. In a follow-up study, three-weeks of qBWSTT was also shown not to attenuate trabecular bone deterioration after SCI [59].

Based on the evidence that is presented above, it remains unknown whether ABPTs that reload the impaired limbs after SCI alter bone turnover in a manner that is sufficient to improve BMD. With the above considered, the next sections summarize the findings of clinical studies that met the following criteria: (1) enrolled adults with incomplete and/or complete SCI and (2) reported BMD at the distal femur or proximal tibia, or BMD or T-scores at other sublesional sites, both before and after an ABPT intervention regardless of other study characteristics. In doing so, we considered several important questions. First, did the study enroll persons with acute to subacute (0–12-months) SCI when bone loss is the most rapid, or during the chronic (>12-month) period when bone loss is more gradual? Secondly, did the study assess the bone changes at the relevant fracture-prone sites after SCI (i.e., distal femur or proximal tibia) or at other bone sites? Thirdly, did the study report that an ABPT attenuated bone loss or increased BMD? We considered trials that reported attenuated bone loss or increased BMD vs. the baseline or a control group to have demonstrated improvement, while those not meeting these criteria were considered to have shown no effect. For studies that combined an ABPT with a pharmacologic intervention, we only discuss the groups that were receiving ABPT alone. Case studies, case series, and cross-sectional studies that did not statistically assess the pre-post differences are discussed from a qualitative perspective.

## 10. ABPT Interventions and BMD after Acute/Subacute SCI

**Figure 1:**
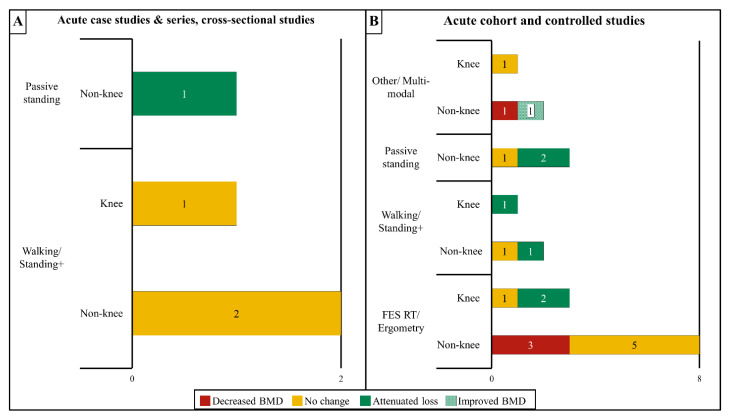
Summary of the effects of activity-based physical therapy (ABPT) on changes in bone mineral density (BMD) at the knee (distal femur and proximal tibia) or all other sublesional non-knee sites in persons with acute/sub-acute SCI for (**A**) case studies, case series, and cross-sectional studies or (**B**) controlled and cohort studies. The total number of studies that reported decreased BMD, no BMD change, attenuated BMD loss, or improved BMD after an ABPT modality are provided in the corresponding bars. Some studies examined both knee and non-knee sites – in these instances, the individual results for knee and non-knee sites were included in each bar. If a study compared more than one modality, the individual results for each modality were included if available. If no studies were found that fit a certain category, the category was omitted from the chart. Overall, most acute studies showed no effect or attenuated BMD loss over time. FES, functional electric stimulation; RT, resistance training; “Standing+” refers to interventions that combined standing modalities with other modalities that increase muscle activation such as FES or vibration.

A visual summary of the studies examining the effects of different ABPT modalities at knee and non-knee sites in acute and subacute SCI is presented in Figure 1A,B. Case studies or case series that enrolled persons in the acute to subacute post-SCI period utilized BWSTT with manual- [50] or robotic-assistance [38], or FES rowing [106]. Of these, none reported increased BMD vs. baseline. Only FES rowing appeared to attenuate the distal femur BMD loss when compared with the expected bone loss rate during the subacute SCI phase [106], while neither BWSTT with manual- [50] or robotic-assistance [38] produced an apparent effect on BMD (Table 1). Using a cross-sectional design, Goemaere et al. [107] also reported that those with incomplete or complete SCI who initiated passive standing during the acute SCI phase displayed less aBMD deficits at the lumbar spine [0% difference (standing) vs. −7.4% (non-standing)] and femoral shaft [−21% (standing) vs. −29.2% (non-standing)] when compared with normative aBMD values that were derived from a non-SCI cohort, although, standing did not appear to attenuate aBMD deficits at the total hip or femoral neck or trochanter.

No trial without a control comparator [103,108] nor any controlled trial [28,31,51,99,109,110,111,112,113,114,115] that enrolled persons in the acute/subacute period reported increased sublesional BMD vs. the baseline, irrespective of the site that was evaluated (Table 2). There were two uncontrolled trials that reported no obvious attenuation of sublesional bone loss after ABPT. Rodgers et al. [108] reported that distal tibia trabecular vBMD was 5.4% lower vs. the baseline in a cohort of persons with subacute or chronic SCI involved in a 12-week FES RT protocol, with the subacute SCI participant displaying the greatest bone loss (−26% vs. the baseline) among all the participants. Astorino et al. [103] also reported that a cohort of persons with acute/subacute SCI or chronic SCI exhibited progressively lower aBMD at most sites that were examined, including the distal femur and proximal tibia, while undergoing a six-month multimodal ABPT regimen that involved lower extremity RT, BWSTT, overground walking, vibration training, and FES cycling. Similarly, 5 of the 11 controlled trials did not detect attenuated sublesional BMD loss at any skeletal site that was examined [51,99,110,111,114]. These trials ranged from 6-weeks to 12-months in duration (training frequency three to five days/week) and used the following modalities: passive unilateral standing [110], BWSTT with standing [114], and FES-based RT [51,99,111]. Of these, only Groah et al. [51] examined BMD at the knee.

In comparison, 6 of the 11 controlled trials reported that an ABPT attenuated BMD loss at different sublesional sites [28,31,109,112,113,115]. Lai et al. [31] reported that 3-months of FES cycling attenuated aBMD loss at the distal femur (−2.2% from the baseline) as compared to the controls (−6.7% from the baseline). Dudley-Javoroski et al. [113] reported that FES-mediated quadriceps contractions attenuated distal femur vBMD loss in the limb that was undergoing unilateral standing (25% less bone loss at 1-year and 39% less bone loss at three-years vs. the non-stimulated limbs), with a follow-up analysis confirming that FES attenuated trabecular vBMD loss in a roughly similar pattern in the anterior and posterior distal femur quadrants [116]. Shields et al. [28] also reported ~50% less proximal tibia aBMD loss in the leg that underwent >1.6-years of NMES (−17% from the baseline) vs. the untrained leg of the same participant (−32% from the baseline). Additionally, three controlled trials reported attenuated BMD loss at sites other than the knee. Shields and Dudley-Javoroski [115] reported a 31% higher trabecular vBMD at the distal tibia epiphysis in the limb that received FES strengthening 5 days/week for two- to three-years vs. the untrained limb and De Bruin et al. [112] reported that BWSTT or use of standing frames lessened distal tibia trabecular vBMD loss over six-months (−0.4% from the baseline for both interventions) vs. the controls (−8% from the baseline). Lastly, Alekna et al. [109] reported that passive standing (>one hour/day, five days/week, for two-years) lessened aBMD loss vs. controls within the whole leg (−25% vs. −34% from the baseline), and pelvis (−15% vs. −21% from the baseline). Regardless, five of six controlled trials that observed attenuated BMD loss at the sites that were described above also reported no BMD differences in the treatment vs. control groups at other sites [28,31,109,112,115]. Of note, four of six trials that reported attenuated BMD loss utilized FES exercises [28,31,113,115] and two used standing exercise without FES [109,112]. However, a similar number of FES [51,99,111] and standing trials [110,114] observed no improvement in BMD at any site that was evaluated.

## 11. ABPT Interventions and BMD after Chronic SCI

**Figure 2:**
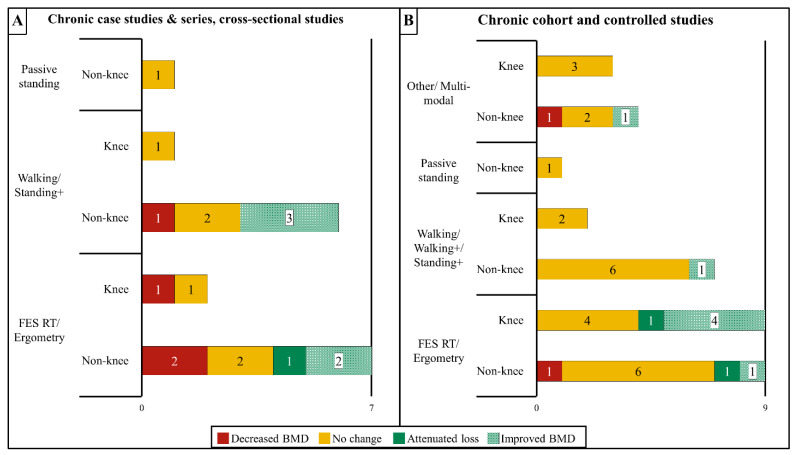
Summary of the effects of activity-based physical therapy (ABPT) on the changes in bone mineral density (BMD) at the knee (distal femur and proximal tibia) or all other sublesional non-knee sites for persons with chronic SCI in (**A**) case studies, case series, and cross-sectional studies, or (**B**) controlled and cohort studies. The data are reported as described in Figure 1A,B. Overall, most studies on persons with chronic SCI reported that ABPT did not alter BMD with only four studies reporting improved BMD at the knee. FES, functional electric stimulation; RT, resistance training; “Walking+” refers to interventions that combined walking ABPT with FES or nerve stimulation; “Standing+” refers to interventions that combined standing modalities with other modalities that increase muscle activation such as FES or vibration.

A visual summary of studies examining the effects of different ABPT modalities at knee and non-knee sites in chronic SCI is presented in Figure 2A,B. Case studies or case series that enrolled participants with chronic SCI ranged from 10-weeks to 8-years in duration, instituted training frequencies of two- to five-days/week, and utilized the following modalities (Table 3): passive standing in a frame that was combined with leg or whole-body vibration [117], overground walking that was assisted by reciprocating gait orthosis [118], BWSTT alone [119] or in combination with epidural electrical stimulation [120] or nerve stimulation [121], or FES-based cycling [122,123] or rowing [124] that was delivered alone or after FES RT [106,125,126]. These reports produced inconsistent results with four case studies [117,121,123,125] and one case series [118] observing an apparent BMD increase ranging from 2–20% vs. the baseline, three case studies [121,122,124] and one case series [120] reporting no clear bone change (<2% change vs. baseline), and two case studies [117,119] and three case series [106,118,126] observing an apparent BMD reduction ranging from 2–21% vs. the baseline, depending on the site that was evaluated. Goktepe et al. [127] also reported no differences in T-scores among persons with chronic SCI who performed ≥one hour/day or <one hour/day standing or no standing at several traditional osteoporosis sites. Of these studies, only two analyzed BMD changes at the knee. Lambach et al. [106] reported distal femur trabecular vBMD was ~13% lower in two persons with chronic SCI after 9–12 months of FES RT and rowing, while Coupaud et al. [121] reported inconsistent distal femur and proximal tibia total and trabecular vBMD changes (range: −2.8% to +2.5% vs. the baseline) in a person with chronic SCI who underwent BWSTT with unilateral peroneal nerve stimulation for seven-months. Within a cross-sectional design, Gibbons et al. also reported that a person with chronic SCI displayed 31% higher proximal tibia trabecular vBMD after completing eight-years of FES rowing [124] and 80–125% higher total and trabecular vBMD at the distal tibia after 10-years, [15] when compared with a historical cohort of persons with chronic SCI, although, vBMD remained 7–19% lower in this person compared with uninjured persons. Of note, three of five case studies/series that reported higher BMD vs. a baseline incorporated FES [123,125] or nerve stimulation [121], while four others that utilized FES reported no BMD change [122,124] or reduced BMD vs. the baseline [106,126].

Inconsistent results also exist in uncontrolled and controlled trials that evaluated the effects of ABPT on bone in persons with chronic SCI. In total, we identified 14 trials without a control comparator group (Table 4), which ranged from 6-weeks to 16-months in duration with a training frequency of two- to seven-days/week and assessed: passive loading in a standing frame [128], standing that was combined with whole body vibration [102], BWSTT alone [100], over ground walking in an exoskeleton [129] or reciprocating gait orthosis [130], a multimodal ABPT regimen (described above) [103], or FES-based overground walking [131], RT [108], cycling [29,30,132], or a combination of RT and cycling [133,134,135]. Three uncontrolled trials reported increased BMD [29,30,134] vs. a baseline at some sublesional sites, while 10 reported no bone change at any sublesional site evaluated [100,102,128,129,130,131,132,133,134,135] and one enrolling persons with acute and chronic SCI observed BMD reductions at most sites assessed [103]. In addition, we identified 10 controlled trials (Table 5) that ranged from 12-weeks to 12-months in duration, utilized a training frequency of three- to five-days/week and evaluated: seated vibration to the lower limbs [101,136], a combined protocol involving BWSTT, overground walking, and RT [97], or FES while undergoing BWSTT [98], RT [137,138], cycling [139], or RT and cycling [96,140] or rowing [141]. A total of two controlled trials reported increased BMD vs. a baseline at select bone sites [96,137] and two indicated higher BMD vs. a control group [97,140], while five reported no difference vs. a baseline or a control group at any site evaluated [98,101,136,138,139] and one acquired but did not report BMD [141].

In total, five uncontrolled [13,29,30,100,103] and nine controlled trials [96,98,101,136,137,138,139,140,141] evaluated distal femur and/or proximal tibia BMD. Most of these studies reported neither an attenuation of BMD loss nor increased BMD at areas surrounding the knee [96,98,100,101,103,136,138,139,141]. However, a small subset of these trials reported improved BMD at the knee, with each study using FES-based RT and/or cycling [29,30,134,137,140]. For example, the distal femur and/or proximal tibia aBMD increased 10–15% vs. the baseline in studies that utilized 6–18 months of FES knee extension RT (distal femur: +18%, proximal tibia: +15%) [137] or cycling (distal femur: +11%, proximal tibia: +10–13%) [29,30]. Similarly, the total and trabecular vBMD at the distal femur were improved 7% to 13% vs. the baseline, respectively, in a study that utilized three-months of FES knee extension RT followed by nine-months of FES cycling [134], suggesting that trabecular bone responds more robustly to FES than cortical bone. In this regard, Hangartner et al. reported that 12-weeks of FES knee extension RT followed by 12- to 36-weeks of FES cycling produced an estimated 3.3% less proximal tibia trabecular vBMD loss per year when compared with non-exercised controls, while no apparent cortical vBMD improvement existed [140]. A total of two trials that incorporated bodyweight-supported [97] or full weight-bearing activity without FES [103] also observed an increased aBMD at sites other than the knee. Mobarake et al. [97] reported that a 12-week BWSTT regimen increased the lumbar spine and femoral neck aBMD by +9% and +17% from the baseline, respectively, vs. a +1.2–1.3% change for the controls. Similarly, Astorino et al. [103] reported that a six-month multimodal ABPT regimen increased lumbar spine aBMD by +4.7% vs. the baseline. However, as indicated above, seven trials that assessed FES-based modalities [108,132,133,135,138,139,141] and several that incorporated standing without FES [102,128] or exoskeleton/assisted walking/BWSTT without FES [100,129,130] reported no BMD improvement, irrespective of the site that was examined.

## 12. Common Parameters to Improve BMD after SCI

Several common parameters existed among studies that reported improved proximal tibia or distal femur BMD or increased BMD at other sites. First, studies that reported attenuated BMD loss at the knee all enrolled persons with acute/subacute SCI and used FES-based modalities that were performed 3–5 days/week, 20–60 min/day, for ≥3-months [28,31,106,113] and studies reporting increased BMD at the knee all enrolled persons with chronic SCI and used FES-based regimens performed 3–5 days/week, 30+ min/day, for ≥6-months [29,30,134,137]. Second, no study that incorporated standing without FES observed improvements in the distal femur or proximal tibia BMD, although, some that utilized these regimens 3–7 days/week, 60+ min/day, for >3-months reported attenuated BMD loss [107,109,112] or increased BMD at other sites [97,103]. These observations highlight the need to target ABPTs to the specific region(s) where BMD improvements are most warranted and to use a training intensity, frequency, and duration that is sufficient to improve BMD. Regardless, it is important to reiterate that not all FES modalities or passive/active standing regimens that met these criteria produced BMD improvements. For example, Clark et al. [111] and Arija-Blazquez et al. [99] reported that FES-based RT regimens lasting 14-weeks to 5-months (performed 5-days/week for 30+ min/day) did not attenuate sublesional aBMD loss in persons with acute/subacute SCI and other FES studies that met the chronic criteria reported no BMD improvement at the distal femur or proximal tibia [96,138,139], or at other skeletal sites [108,131,135].

Several other factors that were associated with BMD improvements were also identified. First, higher total work output was associated with greater BMD gain. For example, Lambach et al. [106] reported that a higher magnitude of loading and more weekly training sessions were both associated with less trabecular vBMD loss at the proximal femur in persons that were undergoing FES rowing, and Draghici et al. [142] reported that total distance rowed and peak foot reaction force were positively associated with the preservation of the distal tibia trabecular thickness in persons that were undergoing FES rowing, when these factors and SCI duration were incorporated into stepwise regression models. Similarly, Bloomfield et al. [96] reported that distal femur aBMD was improved (+17.8% vs. the baseline) in a subgroup of persons who achieved power outputs >18 watts (W) during FES cycling, while aBMD was not improved in those with lower power output (<12 W, BMD change +0.2%) or in non-exercising controls (−2.3% vs. the baseline) and Frotzler et al. [134] reported that FES cycling improved the total and trabecular vBMD at the distal femur with participants averaging ~18 W power output. Regardless, 18 W may be difficult to sustain for many persons with SCI. As evidence, Eser et al. [143] reported that power outputs ranged from 10.8 to 13.6 W in persons with acute SCI who completed an average of 47 FES cycling bouts, with higher FES frequency producing higher power outputs. Of these participants, less than one-third achieved an average power output >18 W. Second, a continued training stimulus is required to maintain BMD if skeletal improvements are achieved. As evidence, in two studies that reported FES increased aBMD vs. the baseline, a regression of BMD to the baseline occurred after a six-month period involving no FES cycling [29] or once weekly FES [30].

## 13. Future Directions

As discussed above, only limited evidence supports the notion that ABPTs attenuate BMD loss in persons with acute/subacute SCI or increase BMD in the paralyzed limbs after chronic SCI, especially when considering the most fracture-prone sites that surround the knee. Moreover, few studies have evaluated whether ABPTs improve trabecular or cortical bone microstructure at the distal femur or proximal tibia (Table 6) and none have assessed whether the potential BMD gains or bone microstructural changes mitigate the fracture risk. Interestingly, Fang et al. [144] and Edwards et al. [101] both used FEA to predict the bone mechanical characteristics in response to 12-months of FES rowing or seated vibration, respectively, and observed no tensile or compressive strength improvements at the distal femur or proximal tibia. However, neither study observed BMD or bone microstructural changes at areas surrounding the knee in response to the respective modalities. Given these observations, we suggest future research focuses on optimizing the FES parameters to improve the distal femur and proximal tibia bone parameters, with emphasis on the following questions:

- What are the optimal FES and modality-specific parameters (e.g., stimulation frequency, amplitude, pulse width, power output, tibio-femoral strain, and pedaling cadence) to stimulate and/or maintain bone improvements?
- What is the optimal training frequency (number of sessions per day or per week) and training duration (time per bout and intervention length) to improve bone parameters?
- What is the minimum training frequency and duration to maintain bone after SCI?
- Is the magnitude of BMD or bone microstructural gains that result from FES sufficient to improve bone strength or bone mechanical characteristics and/or to reduce the fracture risk at the distal femur and/or proximal tibia after SCI?

Clinical trials focused on optimizing FES would require a large, concerted effort over many years to discern optimal parameters, due to the relatively slow bone remodeling in humans and the number of potential parameter combinations. To lessen this burden, percutaneous ES [105,145,146] or direct nerve stimulation [64,104,147] techniques could be used to optimize the FES parameters and to identify the optimal training frequency and duration in preclinical SCI models, with only the most successful protocols advancing. Preclinical SCI models can also be used to evaluate combinatory strategies to improve BMD. In this regard, several recent preclinical and clinical studies have assessed ABPTs combined with antiresorptive drugs, such as zoledronic acid [141,144] or testosterone [59,104,148], or bone anabolic drugs such as teriparatide [101], with varying success.

Regardless of the intervention that is used, we suggest the following practices be implemented when evaluating bone changes after SCI. First, studies should quantify the distal femur and/or proximal tibia bone parameters before and after an intervention using an established imaging modality (e.g., DXA or pQCT) and a validated protocol, instead of assessing surrogate bone sites. We recognize that DXA is used to quantify whole-body fat-free mass or fat mass changes, and thus, aBMD that is assessed in the whole-body or at other traditional osteoporosis sites may be reported out of convenience. Future work should explain the relevance of BMD changes at these traditional osteoporosis sites in relation to the more fracture-prone distal femur and proximal tibia sites after SCI. Second, clinical trials should last a minimum of 6–12 months with ABPT performed ≥3 days/week for ≥30 min/day across the intervention, given the slow bone turnover and BMD improvements that are present in humans in response to loading. Third, a control group or control comparator (e.g., untrained limb) should be assessed to ensure the appropriate data interpretation. Fourth, interventions should attempt to quantify the tibio-femoral forces during training, as has been previously reported [149], and/or attempt to achieve average power outputs >18 W when utilizing FES cycling as regimens exceeding this threshold appear to have a higher likelihood to improve BMD [96]. Fifth, studies should assess the fracture risk and/or evaluate the bone tensile properties via FEA or another method, if available, to establish ABPT effectiveness because the reduction in bone strength over the first few months after SCI has been estimated to be three times greater than the aBMD loss as determined by DXA [36] and because any given percentage increase in whole-bone BMD resulting from loading has been associated with 10 times greater percentage increase in bone strength in preclinical models [90], suggesting that BMD changes do not reflect the bone mechanical properties that may influence non-traumatic fracture risk.

## 14. Conclusions

**Figure 3:**
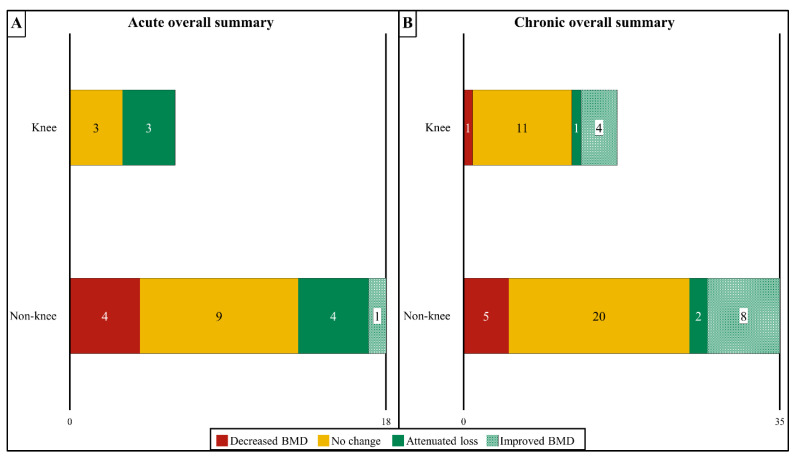
Summary of the effects of activity-based physical therapy (ABPT) on changes in bone mineral density (BMD) at the knee (distal femur and proximal tibia) or all other sublesional non-knee sites for persons with acute/sub-acute (**A**) or chronic (**B**) SCI in aggregate. Overall, half of all acute studies and most chronic studies showed no effect on BMD at the knee or non-knee sites. For data on specific ABPT modalities see Figure 1A,B (acute/subacute SCI) or Figure 2A,B (chronic SCI).

A visual summary of all the reviewed studies examining the effects of ABPT modalities at knee and non-knee sites in acute and chronic SCI is presented in Figure 3A,B. Various ABPTs promote neuromuscular benefits and use-dependent neuroplasticity in persons with SCI [78]. Despite this, no known ABPT completely prevents bone loss that develops in the lower extremities over the acute/subacute post-SCI period, regardless of the skeletal site that is evaluated. Moreover, no ABPT has shown universal success in increasing BMD at the highly fracture-prone sites surrounding the knee. However, a small subset of trials that evaluated FES modalities reported attenuated BMD loss at the distal femur and/or proximal tibia in persons with acute to subacute SCI and increased BMD in those with chronic SCI, suggesting such regimens hold promise. Additionally, a small subset of studies that utilized weight-bearing ABPTs without FES reported BMD gains at other sites but no BMD changes in areas near the knee. As such, research is needed to understand why ABPTs produce variable BMD responses after SCI and to optimize the training parameters for bone gain. Moreover, routine monitoring of the distal femur and proximal tibia BMD appears important to assess the fracture risk before engaging in ABPTs and to monitor bone changes at these highly fracture-prone sites.

## Figures and Tables

**Table 1:** Case studies, case series, and cross-sectional studies evaluating the effects of activity-based physical therapy (ABPT) and/or loading on bone mineral density (BMD) in adults with acute or subacute spinal cord injury (SCI).

Author; Citation; Sample Size/Sex; Group (G): Modality	SCI Level; Severity; Duration	Training Duration; Frequency; Time; Parameters	Skeletal Site Evaluated and Outcomes Reported	Baseline BMD	BMD (% Difference)		
CASE STUDIES AND CASE SERIES							
		BWSTT					
Giangregorio et al. [50] N = 2 M, 3 F BWSTT	C3-C8; AIS B-C; 66–170 d	6–8 mo (48 sessions); 2 d/wk; Three 5–15 min bouts/d Training parameters: 0–97% BWSTT, 0.6–2.0 km/h bodyweight support reduced, and speed and duration increased over time	Total body aBMD Lumbar spine aBMD Proximal femur aBMD Distal femur aBMD Proximal tibia aBMD Femur diaphysis vBMD Tibia diaphysis vBMD	Baseline 1.21 g/cm^2^ 1.09 g/cm^2^ 1.03 g/cm^2^ 1.10 g/cm^2^ 0.96 g/cm^2^ 795 g/cm^3^ 796 g/cm^3^	6–8 mo −2.7% −3.1% −2.9% −13% −4.3% −5.5% −6.2%		
Lichy and Groah [38] N = 1 M BWSTT	T4; AIS C; 11 wk	3 mo; 3 d/wk; 1 h/d Training parameters: 16–32 kg BWSTT, 1.9–2.5 km/h, bodyweight support reduced and speed increased over time	Lumbar spine aBMD Left proximal femur aBMD Right proximal femur aBMD	Baseline 1.12 g/cm^2^ 0.92 g/cm^2^ 0.93 g/cm^2^	5 mo −2.8% −11% −4.0%	1.5 yrs +2.5% −19% −5.0%	
		FES					
Lambach et al. [106] N = 4 M FES KE/KF RT + FES rowing	C7-T10; AIS A-B; 10–16 mo	9–12 mo (90 sessions); 3 d/wk; 30–60 min/d Training parameters: FES RT: 0–120 mA, 40 Hz, 450 µs, 5 s on:1 s off FES Rowing: N/R	Entire cohort Distal femur trabecular vBMD Distal tibia trabecular vBMD Acute cohort Distal femur trabecular vBMD Distal tibia trabecular vBMD	Baseline 207 mg/cm^3^ 168 mg/cm^3^ 169 mg/cm^3^ 121 mg/cm^3^	30 sessions −8.4% −14% −8.1% −19%	60 sessions −5.9% −17% −3.9% −21%	90 sessions −8.2% −19% −3.7% −24%
	Note: Lambach et al. included N = 2 <1 yr and N = 2 chronic SCI. Baseline and % difference BMD values are reported separately for the entire cohort and the subacute SCI cohort. Values for the chronic cohort are in Table 3						
CROSS-SECTIONAL STUDIES							
Goemaere et al. [107] N = 42 M, 11 F G1: Standing, N = 34 M, 4 F G2: Non-standing, N = 8 M, 7 F	T-L; complete to incomplete; standing began as early as possible after SCI, usually within 3 mo	12–180 mo; 3–7 d/wk; 1 h/d Training parameters: Long leg braces (N = 20), standing frame (N = 9), or standing wheelchair (N = 9)	G1: L3 aBMD G2: L3 aBMD G1: L4 aBMD G2: L4 aBMD G1: Femur neck aBMD G2: Femur neck aBMD G1: Femur trochanter aBMD G2: Femur trochanter aBMD G1: Total hip aBMD G2: Total hip aBMD G1: Femur shaft aBMD G2: Femur shaft aBMD	Baseline N/R N/R N/R N/R N/R N/R N/R N/R N/R N/R N/R N/R	% uninjured value +0.0% −7.4% † +3.8% −4.7% −25% −25% −27% −30% −30% −31% −21% −29% †		

G, Group; BWSTT, bodyweight-supported treadmill training; FES, functional electrical stimulation; RT, resistance training; KE/KF, knee extension/knee flexion; F, female; M, male; C, cervical; T, thoracic; AIS, American Spinal Injury Association Impairment Scale; SCI Duration: time since SCI in relation to start of intervention; aBMD, areal bone mineral density; vBMD, volumetric bone mineral density; min, minute; h, hour; d, day; wk, week; mo, month; N/R, not reported. The % change was reported in individual papers or was manually calculated from the data in tables and/or figures; † indicates a *p* value of <0.05 between the groups; a lack of symbols indicates no statistical differences that were reported versus the baseline or between the groups.

**Table 2:** Uncontrolled and controlled interventional studies evaluating the effects of activity-based physical therapy (ABPT) and/or loading on bone mineral density (BMD) in adults with acute or subacute spinal cord injury (SCI).

Author; Citation; Sample Size/Sex; Group (G): Modality	SCI Level; Severity; Duration	Training Duration; Frequency; Time; Parameters	Skeletal Site Evaluated and Outcomes Reported	Baseline BMD	BMD (% Difference versus Baseline)			
UNCONTROLLED INTERVENTIONAL TRIALS								
		FES						
Rodgers et al. [108] N = 9 M, 3 F FES KE RT	C4-T10; incomplete and complete; 0.7–17 yr	12 wk (actual 12–18 wk); 3 d/wk; 30 min/d Training parameters: 100 mA, 35 Hz, 300 µs	Total cohort Distal tibia trabecular vBMD Acute participant Distal tibia trabecular vBMD	Baseline 235 mg/cm^3^ 400 mg/cm^3^	12–18 wk −5.4% −26%	Note: vBMD assessed on N = 1 acute and N = 7 chronic SCI. Values = avg of the total and acute cohorts. Chronic cohort data are in Table 4.		
		MULTIMODAL						
Astorino et al. [103] N = 11 M, 2 F Multimodal ABPT regimen	C4-L1; Complete and incomplete; 1.9 ± 2.7 yr (0.2–9.3 yr)	6 mo; >2 d/wk; 2–3 h/d Training parameters: Individualized protocol that included active assistive exercise, lower extremity, and core RT, upper extremity cycle ergometry, bodyweight supported elliptical training, BWSTT, OGW, vibration training, and/or FES cycling	Total body aBMD Lumbar spine aBMD R Total hip aBMD L Total hip aBMD R Femur neck aBMD L Femur neck aBMD R Femur trochanter aBMD L Femur trochanter aBMD R Distal femur aBMD L Distal femur aBMD R Proximal tibia aBMD L Proximal tibia aBMD	Baseline 1.24 g/cm^2^ 1.27 g/cm^2^ 0.96 g/cm^2^ 0.99 g/cm^2^ 0.98 g/cm^2^ 0.99 g/cm^2^ 0.75 g/cm^2^ 0.79 g/cm^2^ 0.92 g/cm^2^ 0.92 g/cm^2^ 0.88 g/cm^2^ 0.89 g/cm^2^	3 mo −1.6% * +3.2% * −3.1% * −4.0% * −2.0% * −3.0% * −2.7% −5.1% * −5.4% −4.4% −3.4% −4.5%	6 mo −2.5% * +4.7% * −6.3% * −7.1% * −5.1% * −4.0% * −6.7% * −8.9% * −11% −7.6% −8.0% −11%	Note: BMD determined on N = 8 <1 yr SCI and N = 5 chronic SCI. Values are avg of the total cohort and were not determined separately for acute and chronic SCI cohorts.	
CONTROLLED INTERVENTIONAL TRIALS								
		BWSTT						
de Bruin et al. [112] N = 13 M G1: BWSTT + standing, N = 4 M G2: Standing, N = 5 M G3: Low activity, N = 4 M	C4-L1; AIS A-D; 1–5 wk	6 mo; 5 d/wk; 60 min/d Training parameters: G1: 20–80% BWSTT (30 min/d), standing (30 min/d), ≥5 h/wk G2: Standing, ≥5 h/wk G3: Loading, 0–5 h/wk	Distal tibia + diaphysis G1: Trabecular vBMD G2: Trabecular vBMD G3: Trabecular vBMD G1: Cortical vBMD G2: Cortical vBMD G3: Cortical vBMD	Baseline N/R N/R N/R N/R N/R N/R	6 mo −0.4% † −0.5% † −8.0% † −0.7% −1.1% −0.6%	Note: † indicates statistically significant difference between G1 and G2 vs. G3, when indicated.		
Frey-Rindova et al. [114] N = 24 M G1: BWSTT + standing, N = 13 M G2: Low activity, N = 11 M	C4-L1; Frankel A-C; 1–4 wk	12 mo; ≥3 d/wk; 30 min/d Training parameters: G1: Partial bodyweight support, ~1.3 km/h G2: Same parameters performed <3 d/wk or <30 min/d	Distal Tibia G1: Trabecular vBMD G2: Trabecular vBMD G1: Cortical vBMD G2: Cortical vBMD	Baseline 316 mg/cm^3^ 302 mg/cm^3^ 935 mg/cm^3^ 910 mg/cm^3^	6 mo −7% −3% −2% −1%	12 mo −8% * −20% * −6% ** −8% **		
		STANDING						
Alekna et al. [109] N = 44 M, 10 F G1: Standing, N = 22 M, 5 F G2: No standing, N = 22 M, 5 F	C2-L1; AIS A-B; 11 ± 3 wk	2 yr; ≥5 d/wk; ≥1 h/d Training parameters: G1: Passive standing G2: No standing	G1: Total body aBMD G2: Total body aBMD G1: Lumbar spine aBMD G2: Lumbar spine aBMD G1: Pelvis aBMD G2: Pelvis aBMD G1: Whole leg aBMD G2: Whole leg aBMD	1.26 g/cm^2^ 1.26 g/cm^2^ 1.28 g/cm^2^ 1.26 g/cm^2^ 1.18 g/cm^2^ 1.18 g/cm^2^ 1.36 g/cm^2^ 1.38 g/cm^2^	1 yr −8.9% * −12% * −11% −14% −12% −15% −20% * −24% *	2 yr −11% * −15% * −14% −17% −15% *† −21% *† −25% *†† −34% *††		
Ben et al. [110] N = 16 M, 4 F G1: Unilateral standing, N = 20 G2: Untrained limb, N = 20	N/R; Nonambulatory; 4 ± 2 mo	12 wk; 36 sessions; 30 min/d Training parameters: G1: Passive unilateral standing G2: No loading	G1: Proximal femur aBMD G2: Proximal femur aBMD	Baseline 0.91 g/cm^2^ 0.91 g/cm^2^	12 wk −6.1% −6.7%			
		FES/NMES						
Arija-Blazquez et al. [99] N = 8 M G1: FES RT, N = 5 M G2: SHAM FES, N = 3 M	T4-T12; AIS A; G1: 5.5 ± 1.1 wk G2: 5.8 ± 1.7 wk	14 wk; 5 d/wk; 47 min/d Training parameters: Both: 8 sets × 10 contractions G1: 0–140 mA, 30 Hz, 200 µs G2: 0 mA, 30Hz, 200 µs	G1: Lumbar spine aBMD G2: Lumbar spine aBMD Femur G1: Total hip aBMD G2: Total hip aBMD G1: Femur neck aBMD G2: Femur neck aBMD G1: Ward triangle aBMD G2: Ward triangle aBMD G1: Trochanter aBMD G2: Trochanter aBMD G1: Intertrochanteric aBMD G2: Intertrochanteric aBMD G1: Whole leg aBMD G2: Whole leg aBMD	Baseline 0.91 g/cm^2^ 1.23 g/cm^2^ 0.92 g/cm^2^ 1.08 g/cm^2^ 0.79 g/cm^2^ 0.96 g/cm^2^ 0.65 g/cm^2^ 0.86 g/cm^2^ 0.70 g/cm^2^ 0.83 g/cm^2^ 1.05 g/cm^2^ 1.22 g/cm^2^ 1.19 g/cm^2^ 1.44 g/cm^2^	14 wk +3.5% +2.1% −7.0% −0.4% −7.6% −8.3% −6.5% −7.7% −9.9% −8.1% −5.9% −1.0% −2.9% −3.3%			
Clark et al. [29] N = 33 (N per sex N/R) G1: FES RT, N = 23 G2: Inactive SCI control, N = 10	C4-T12; AIS A-D; 3 wk	6 mo; 5 d/wk; 30 min/d Training parameters: G1: 2 × 15 min/d; 4:8 s on:off G2: Inactive control	G1: Total body aBMD G2: Total body aBMD G1: Lower extremity aBMD G2: Lower extremity aBMD G1: Femur neck aBMD G2: Femur neck aBMD G1: Femur proximal aBMD G2: Femur proximal aBMD	Baseline N/R N/R N/R N/R N/R N/R N/R N/R	3 mo −2.2% †† +0.7% †† −2.4% −2.3% −4.5% −3.2% −6.0% −3.7%	6 mo −3.0% −1.9% −7.1% −4.7% −11.6% −6.5% −11% −8.4%		
Dudley-Javoroski et al. [113] N = 24 M, 9 F G1: FES standing, N = 6 M, 1 F G2: Passive standing, N = 5 M G3: No standing, N = 13 M, 3 F	C5-T12; AIS A-B; G1: 0.2–2.1 yr G2: 0.2–0.7 yr G3: 0.2–24 yr Note: G2 had N = 5 acute and N = 2 chronic SCI	3 yr; 5 d/wk; 30 min/d Training parameters: G1: FES 10 min/d while unilateral standing, 0–200 mA, 20 Hz, 200 µs, 5:5 s on:off (2 bouts × 60 100-pulse trains), produced 150% bodyweight compressive load G2: 40% bodyweight compressive load G3: 0% BW (no standing)	G1: Distal femur vBMD G2: Distal femur vBMD G3: Distal femur vBMD G1: Proximal tibia vBMD G1: Proximal tibia vBMD G1: Proximal tibia vBMD G1: Distal tibia vBMD G2: Distal tibia vBMD G3: Distal tibia vBMD	Baseline N/R N/R N/R N/R N/R N/R N/R N/R N/R	1 yr vs. G2 +30% † +23% +13%	1 yr vs. G3 +48% † +29% −11%	3 yr vs. G2 +41% † +21% −14%	3 yr vs. G3 +102% † +47% −37%
Dudley-Javoroski et al. [116] N = 11 M, 1 F G1: FES standing, N = 6 M, 1 F G2: Passive standing, N = 5 M	C5-T12; AIS A-B; G1: 0.2–2.1 yr G2: 0.2–0.7 yr	3 yr; 3 d/wk; 30 min/d Training parameters: G1: FES 10 min/d while unilateral standing, 0–200 mA, 20 Hz, 200 µs, 5:5 s on:off (2 bouts × 60 100-pulse trains), produced 150% bodyweight compressive load G2: 40% bodyweight compressive load	Femur distal (trabecular) G1: Antero-lateral vBMD G2: Antero-lateral vBMD G1: Antero-medial vBMD G2: Antero-medial vBMD G1: Postero-lateral vBMD G2: Postero-lateral vBMD G1: Postero-medial vBMD G2: Postero-medial vBMD	Baseline 233 mg/cm^3^ 233 mg/cm^3^ 188 mg/cm^3^ 188 mg/cm^3^ 220 mg/cm^3^ 220 mg/cm^3^ 224 mg/cm^3^ 224 mg/cm^3^	0.25- 0.50 yr −1.6% −22% +3.8% −25% +3.3% −20% +6.4% −15%	0.50- 0.75 yr −3.5% −19% −1.8% −20% −0.5% −12% +2.4% −9%	0.75- 1.0 yr −0.6% −24% +2.3% −26% −4.8% −21% +0.9% −15%	1.0- 1.5 yr −12% −19% −9.2% −17% −12% −23% −6.3% −14%
Groah et al. [51] N = 22 M, 4 F G1: FES RT, N = 15 M, 1 F G2: SCI control, N = 7 M, 3 F	>T12; AIS A-B; 35.9 ± 23.3 d	6 wk; 5 d/wk; 1 h/d Training parameters: G1: 0–125 mA, 25 Hz, 300 µs, 5:5 s on:off G2: Standard of care control	G1: Lumbar spine aBMD G2: Lumbar spine aBMD G1: Total hip aBMD G2: Total hip aBMD G1: Distal femur aBMD G2: Distal femur aBMD G1: Proximal tibia aBMD G2: Proximal tibia aBMD	Baseline 1.32 g/cm^2^ 1.27 g/cm^2^ 1.19 g/cm^2^ 1.19 g/cm^2^ 1.11 g/cm^2^ 0.96 g/cm^2^ 1.04 g/cm^2^ 0.86 g/cm^2^	6 wk +1.2% −7.2% −1.9% −13% −4.0% −3.5% −0.9% −4.6%	F/U 3 mo −1.3% −1.9% −15% −12% −7.4% −15% −12% −17%		
Lai et al. [31] N = 20 M, 4 F G1: FES cycling, N = 10 M, 2 F G2: SCI control, N = 10 M, 2 F	C5-T9; AIS A; 26–51 d	3 mo; 3 d/wk; 30 min/d Training parameters: G1: mA controlled by microprocessor, 20 Hz, 300 µs G2: Control	G1: Femur neck aBMD G2: Femur neck aBMD G1: Distal femur aBMD G2: Distal femur aBMD	Baseline 0.93 g/cm^2^ 0.91 g/cm^2^ 1.00 g/cm^2^ 1.00 g/cm^2^	3 mo −4.6% * −5.1% * −2.2% *† −6.7% *†	F/U 3 mo −9.1% # −9.8% # −9.0% # −14% #		
Shields et al. [28] N = 6 (sex N/R) G1: NMES trained limb, N = 6 G2: Untrained limb, N = 6	C5-T10; AIS A; 0.16–0.35 yr	1.65–3.0 yr; 5 d/wk; 20 min/d Training parameters: G1: 4 bouts × 120 trains (10 pulse train, 15 Hz, 667 ms every 2 s), 140% bodyweight compressive force on tibia G2: No NMES	G1: Hip aBMD G2: Hip aBMD G1: Proximal tibia aBMD G2: Proximal tibia aBMD Both: Lumbar spine aBMD	Baseline 0.97 g/cm^2^ 0.96 g/cm^2^ 0.29 g/cm^2^ 0.31 g/cm^2^ 1.10 g/cm^2^	1.5–6 mo −14% −13% +4% † −8% † −12%	6–12 mo −22% # −22% # −4% #† −17% #† −14%	12–18 mo −36% # −31% # −3% #† −27% #† −13%	18–36 mo−35% #−34% #−17% #†−32% #†−15%
Shields & Dudley-Javoroski [115] N = 7 (sex N/R) G1: NMES trained limb G2: Untrained limb	C5-T10; AIS A; <6 wk	2–3 yr; 5 d/wk; 35 min/d Training parameters: G1: 0–200 mA at 400 V, 15 Hz, 667 ms, 4 bouts × 10-pulse train every 2 s G2: No NMES	Tibia Diaphysis G1: Cortical vBMD G2: Cortical vBMD Tibia Distal Epiphysis G1: Trabecular vBMD G2: Trabecular vBMD	Baseline N/R N/R N/R N/R	2–3 yr % untrained limb N/R–Not different N/R–Not different +31% vs. untrained			

G, group; BWSTT, bodyweight-supported treadmill training; RT, resistance training; NMES, neuromuscular electrical stimulation; FES, functional electrical stimulation; F, female; M, male; C, cervical; T, thoracic; L, lumbar; AIS, American Spinal Injury Association Impairment Scale; SCI Duration, time since SCI in relation to intervention; aBMD, areal bone mineral density; vBMD, volumetric bone mineral density; min, minute; h, hour; d, day; wk, week; mo, month; yr, year; N/R, not reported; F/U, follow-up after intervention complete; Note: % change was reported in individual papers or was manually calculated from data in tables and/or figures; * indicates <0.05, ** <0.01 vs. the baseline; # indicates <0.05 vs. the initial BMD assessment after the baseline; † indicates a *p*-value of <0.05, †† <0.01 between the marked groups; a lack of symbols indicates no statistical differences that were reported versus the baseline or between groups.

**Table 3:** Case studies, case series, and cross-sectional studies evaluating the effects of activity-based physical therapy (ABPT) and/or loading on bone mineral density (BMD) or T-scores in adults with chronic spinal cord injury (SCI).

Author; Citation; Sample Size/Sex; Group (G): Modality	SCI Level; Severity; Duration	Training Duration; Frequency; Time; Parameters	Skeletal Site and Outcome Reported	Baseline BMD/T-Score	BMD (% Difference)/T-Score (Actual Change)			
CASE STUDIES AND CASE SERIES								
		STANDING/VIBRATION						
Davis et al. [117] N = 1 F Phase 1: Standing Phase 2: Standing + seated vibration Phase 3: Standing + standing vibration	T10; N/R; 4 yr	Three 10 wk phases with 7 wk break between phases; 3 d/wk Training parameters: Phase 1: Standing 40 min/d Phase 2: Standing 20 min/d + vibration (seated) 20 min/d Phase 3: Standing + vibration (standing) 7 min/d Vibration: 30–50 Hz, 2.16–5.83 g	Phase 1: Trunk aBMD Phase 1: Whole spine aBMD Phase 1: Pelvis aBMD Phase 2: Trunk aBMD Phase 2: Whole spine aBMD Phase 2: Pelvis aBMD Phase 3: Trunk aBMD Phase 3: Whole spine aBMD Phase 3: Pelvis aBMD	Baseline 0.92 g/cm^2^ 1.21 g/cm^2^ 0.99 g/cm^2^ 0.89 g/cm^2^ 1.19 g/cm^2^ 0.94 g/cm^2^ 0.84 g/cm^2^ 1.12 g/cm^2^ 0.90 g/cm^2^	Post Phase 1 −8.4% ‡ −4.5% −15% ‡ N/A N/A N/A N/A N/A N/A	Post Phase 2 N/A N/A N/A −5.5% ‡ −7.7% ‡ −2.2% N/A N/A N/A	Post Phase 3 N/A N/A N/A N/A N/A N/A +5.5% ‡ +8.3% ‡ +1.9%	
		BWSTT/OGW						
Forrest et al. [119] N = 1 M BWSTT + standing	C6; AIS B; 1 yr	9 mo; 35 sessions (8 wk break) then 62 sessions; 1 h/d Training parameters: 20–60% BWSTT, 0.83 m/s, bilateral standing, bodyweight support reduced over time	Total body aBMD Total hip aBMD Femur neck aBMD Leg aBMD	Baseline 1.30 g/cm^2^ 1.05 g/cm^2^ 1.01 g/cm^2^ 1.34 g/cm^2^	9 mo −1.5% −21% −18% −6.7%			
Ogilvie et al. [118] N = 2 M, 2 F RGO walking	Level N/R; Paraplegia; >1 yr	24–30 mo; 5 d/wk; 3 h/d	Femur neck vBMD	Baseline N/R	6 mo −5.9%	18 mo −1.9%	24 mo +4.2%	30 mo +1.1%
		FES, EES, or NMES						
Beck et al. [120] N = 2 M BWSTT + OGW + EES	T3-T6; AIS A; >3 yr	18 mo (6 mo without EES + 12 mo with EES); 3 d/wk Training parameters: 45 min BWSTT + 30 min OGW + ≤3 h/d of EES enabled exercise at home	Left hip T-score R hip T-score	Baseline −2.4 −2.3	6 mo −0.1 −0.1	12 mo +0.1 +0.0		
Coupaud et al. [121] N = 1 M BWSTT + unilateral peroneal nerve stimulation to left (L) side	T6; AIS C 14.5 yr	7 mo; 2–3 d/wk; 15–30 min/d Training parameters: 30% BWSTT Unilateral FES (L side) peroneal nerve: 40 mA, 40 Hz, 117–351 µs	Femur (vBMD) R diaphysis cortical L diaphysis cortical R distal epiphysis total L distal epiphysis total R distal epiphysis trabecular L distal epiphysis trabecular Tibia (vBMD) R proximal epiphysis total L proximal epiphysis total R proximal epiphysis trabecular L proximal epiphysis trabecular R diaphysis cortical L diaphysis cortical R distal epiphysis total L distal epiphysis total R distal epiphysis trabecular L distal epiphysis trabecular	Baseline 1103 mg/cm^3^ 1108 mg/cm^3^ 198 mg/cm^3^ 191 mg/cm^3^ 159 mg/cm^3^ 147 mg/cm^3^ 136 mg/cm^3^ 131 mg/cm^3^ 83.1 mg/cm^3^ 85.5 mg/cm^3^ 1122 mg/cm^3^ 1119 mg/cm^3^ 184 mg/cm^3^ 169 mg/cm^3^ 118 mg/cm^3^ 108 mg/cm^3^	7 mo +0.5% +0.3% +1.2% +1.1% +2.2% +0.5% +0.0% +2.5% +1.1% −2.8% −0.3% −0.3% +2.1% ‡ +13% ‡ +4.9% ‡ +20% ‡			
Deley et al. [125] N = 1 F FES RT + FES rowing	T4-T5; AIS A; 2 yr	12 mo (3 mo RT, 9 mo rowing); 3 d/wk; 30 min/d Training parameters: RT: 0–110 mA, 40 Hz, 450 µs, 6:6 s on:off Rowing: 40 Hz, 450 µs	Femur neck aBMD	Baseline 0.53 g/cm^2^	12 mo +19%			
Dolbow et al. [123] N = 1 F FES cycling	T6; AIS A; 2 yr	12 mo; 3 d/wk; 1 h/d Training parameters: 140 mA, 33.3–50 Hz, 250–300 µs, 0.64–1.28 Nm avg resistance, 36–43 rpm	Total body aBMD	Baseline 0.93 g/cm^2^	12 mo +9.5%			
Dolbow et al. [122] N = 1 M FES cycling	C4; AIS C; 33 yr	56 mo; 3 d/wk; 40–60 min/d Training parameters: 120–140 mA, 33.3–35.7 Hz, 250–300 µs, 0.5–2.0 Nm avg resistance, 40–50 rpm	Total body aBMD	Baseline 1.02 g/cm^2^	56 mo −0.6%			
Gibbons et al. [124] N = 1 M FES rowing, N = 1 M	G1: T4; AIS A; 13.5 yr	>8 yr; 2–4 d/wk; 15–45 min/d Training parameters: 0–115 mA, 50 Hz, 450 µs	Total hip T-score	T-score −2.3	>8 yr +0.6	Note: Proximal tibia trabecular vBMD values are reported in the cross-sectional study section below.		
Lambach et al. [106] N = 4 M FES KE/KF RT + FES rowing	C7-T10; AIS A-B; 10–16 mo	9–12 mo (90 sessions); 3 d/wk; 30–60 min/d Training parameters: FES RT: 0–120 mA, 40 Hz, 450 µs, 5 s on:1 s off FES Rowing: N/R	Entire cohort Distal femur trabecular vBMD Distal tibia trabecular vBMD Chronic cohort Distal femur trabecular vBMD Distal tibia trabecular vBMD	Baseline 207 mg/cm^3^ 168 mg/cm^3^ 245 mg/cm^3^ 215 mg/cm^3^	30 sessions −8.4% −14% −8.8% −8.8%	60 sessions −5.9% −17% −7.9% −13%	90 sessions −8.2% −19% −13% −14%	
	Note: Lambach et al. included N = 2 <1 yr and N = 2 chronic SCI. Baseline and % difference values are reported for the entire cohort and the chronic SCI cohort. BMD values for the subacute cohort are in Table 1.							
Pacy et al. [126] N = 4 M FES KE RT + FES cycling	T4-T6; AIS A; 1–8 yr	10 wk RT; 5 d/wk; 15 min/d then 32 wk cycle; 5 d/wk; 15 min/d Training parameters: FES RT: 65–90 V, 40 Hz, 300 µs, 6:6 s on:off FES Cycle: 80–125 V, 50 rpm, 0–18.75 W	Distal tibia vBMD	Baseline 0.16 g/cm^2^	42 wk −3.2%			
CROSS-SECTIONAL STUDIES								
		STANDING						
Goktepe et al. [127] N = 60 M, 11 F G1: Standing ≥1 h/d, N = 15 M, 5 F G2: Standing <1 h/d, N = 9 M, 2 F G3: No standing, N = 36 M, 4 F	Level N/R; AIS A-B; All groups: ≥1 yr (4.41 ± 2.99 yr)	Duration N/R; daily; G1: ≥1 h/d G2: <1 h/d G3: No standing	G1: L2-L4 T-score G2: L2-L4 T-score G3: L2-L4 T-score G1: Femur neck T-score G2: Femur neck T-score G3: Femur neck T-score G1: Ward triangle T-score G2: Ward triangle T-score G3: Ward triangle T-score G1: Femur trochanter T-score G2: Femur trochanter T-score G3: Femur trochanter T-score G1: Total femur T-score G2: Total femur T-score G3: Total femur T-score	Baseline N/R N/R N/R N/R N/R N/R N/R N/R N/R N/R N/R N/R N/R N/R N/R	Actual T-score −0.3 −0.2 +0.1 −1.6 −2.0 −2.0 −1.3 −1.5 −1.5 −2.3 −2.2 −2.5 −2.1 −2.3 −2.4			
		FES						
Gibbons et al. [124] N = 1 M G1: FES rowing, N = 1 M G2: Historical SCI cohort, N = 9 G3: Normative non-SCI, N = 14	G1: T4; AIS A; 13.5 yr G2: C6–T10; AIS A; 6.6 ± 2.8 yr	>8 yr; 2–4 d/wk; 15–45 min/d Training parameters: 0–115 mA, 50 Hz, 450 µs	Proximal tibia trabecular vBMD	Baseline N/R	vs. G2 +31%	vs. G3 −19%		
Gibbons et al. [15] N = 1 M G1: FES rowing, N = 1 M G2: Historical SCI cohort, N = 9 G3: Normative non-SCI, N = 22	G1: T4; AIS A; 14 yr G2: T3–T12; AIS A-B; 11.4 ± 9.4 yr	>10 yr; 3 d/wk; 30 min/d Training parameters: 0–115 mA, 50 Hz, 450 µs	Distal tibia total vBMD Distal tibia trabecular vBMD	Baseline N/R N/R	vs. G2 +82% +125%	vs. G3 −6.5% −15%		

G, group; BWSTT, bodyweight-supported treadmill training; RGO, reciprocating gait orthosis; OGW, overground walking; EES, epidural electrical stimulation; NMES, neuromuscular electrical stimulation; FES, functional electrical stimulation; RT, resistance training; F, female; M, male; C, cervical; T, thoracic; AIS, American Spinal Injury Association Impairment Scale; SCI Duration: time since SCI in relation to intervention reported as range, mean ± SD, or mean and (range); aBMD, areal bone mineral density; vBMD, volumetric bone mineral density; avg, average; min, minute; h, hour; d, day; wk, week; mo, month; yr, year; m, meter; N/R, not reported. Note: % change was reported in individual papers or was manually calculated from data in tables and/or figures; ‡ indicates exceeded least significant change; lack of symbols indicates no statistical differences that were reported versus baseline or between the groups.

**Table 4:** Uncontrolled interventional studies evaluating the effects of activity-based physical therapy (ABPT) and/or loading on bone mineral density (BMD) or T-scores in adults with chronic spinal cord injury (SCI).

Author; Citation; Sample Size/Sex; Modality	SCI Level; Severity; Duration	Training Duration; Frequency; Time; Parameters	Bone Site Evaluated	Baseline BMD/T-Score	BMD (% Difference) T-Score (Actual Change)			
		STANDING/VIBRATION						
Kunkel et al. [128] N = 6 M Standing	C5-T12; Incomplete and complete; 10–39 yr	6 mo (mean ~135 d); 45 min twice daily Training parameters: Standing frame	Lumbar spine aBMD Femur neck aBMD	Baseline 1.26 g/cm^2^ 0.51 g/cm^2^	3 mo −1.6% +18%	6 mo −5.6% +9.8%		
Wuermser et al. [102] N = 5 M, 4 F Standing + vibration	AIS A-B; T3-T12; 2–27 yr	6 mo; 5 d/wk; 20 min/d Training parameters: 0.3 g, 34 Hz sinusoidal movement of 50 µm w/lower extremities supporting ~86% body weight	Total hip aBMD Femur neck aBMD Distal tibia total vBMD Distal tibia trabecular vBMD Distal tibia cortical vBMD	Baseline 0.71 g/cm^2^ 0.75 g/cm^2^ 168 g/cm^3^ 67.5 g/cm^3^ 810 g/cm^3^	3 mo +0.0% −1.3% −2.8% −2.0% −2.6%	6 mo +1.5% +1.4% −3.1% −6.5% −0.9%	F/U 6 mo +2.9% +2.7% −5.0% −7.4% −2.2%	
		BWSTT/OGW						
Giangregorio et al. [100] N = 11 M, 2 F BWSTT	C4-T12; AIS B-C; 1.2–24 yr	12 mo (144 sessions); 3 d/wk; ≤3 bouts of 5–50 min each Training parameters: 0–80% bodyweight support, progressively reduced	Whole body aBMD Lumbar spine aBMD Proximal femur total aBMD Right distal femur total aBMD Right proximal tibia total aBMD Femur diaphysis total vBMD Femur diaphysis cortical vBMD Tibial diaphysis total vBMD Tibial diaphysis cortical vBMD	Baseline 1.12 g/cm^2^ N/R N/R N/R N/R 770 g/cm^3^ 848 g/cm^3^ 745 g/cm^3^ 851 g/cm^3^	12 mo −2.2% N/R −0.2% +1.7% +1.4% −1.6% −0.8% −2.3% −2.0%			
Karelis et al. [129] N = 4 M, 1 F Exoskeleton walking	C7-T10; AIS A; 7.6 ± 4.6 yr	6 wk; 3 d/wk; 60 min/d Training parameters: Walking: 27.0 ± 5.4 min/d Standing: 48.4 ± 7.4 min/d	Total body aBMD Leg aBMD Tibia diaphysis vBMD	Baseline 1.19 g/cm^2^ 1.11 g/cm^2^ 466 mg/cm^3^	6 wk −1.7% +0.5% +14%			
Thoumie et al. [130] N = 6 M, 1 F RGO walking	T2-T10; Severity N/R; 15–60 mo	16 mo; 3 d/wk; 2 h/d Training parameters: RGO walking	Lumbar spine aBMD Femur neck aBMD	Z-score 0.77 1.02	16 mo +0.01 −0.06	Note: Z-score was reported without BMD or T-score values		
		MULTIMODAL						
Astorino et al. [103] N = 11 M, 2 F Multimodal ABPT regimen	C4-L1; Complete and Incomplete; 1.9 ± 2.7 yr (0.2–9.3 yr)	6 mo; >2 d/wk; 2–3 h/d Training parameters: Individualized protocol that included active assistive exercise, lower extremity and core RT, upper extremity cycle ergometry, bodyweight supported elliptical training, BWSTT, OGW, vibration training, and/or FES cycling	Total body aBMD Lumbar spine aBMD R Total hip aBMD L Total hip aBMD R Femur neck aBMD L Femur neck aBMD R Femur trochanter aBMD L Femur trochanter aBMD R Distal femur aBMD L Distal femur aBMD R Proximal tibia aBMD L Proximal tibia aBMD	Baseline 1.24 g/cm^2^ 1.27 g/cm^2^ 0.96 g/cm^2^ 0.99 g/cm^2^ 0.98 g/cm^2^ 0.99 g/cm^2^ 0.75 g/cm^2^ 0.79 g/cm^2^ 0.92 g/cm^2^ 0.92 g/cm^2^ 0.88 g/cm^2^ 0.89 g/cm^2^	3 mo −1.6% * +3.2% * −3.1% * −4.0% * −2.0% * −3.0% * −2.7% −5.1% * −5.4% −4.4% −3.4% −4.5%	6 mo −2.5% * +4.7% * −6.3% *# −7.1% *# −5.1% *# −4.0% * −6.7% *# −8.9% * −11% −7.6% −8.0% −11%	Note: BMD determined on N = 8 <1 yr SCI and N = 5 chronic SCI. Values are an average of the total cohort and were not determined separately for acute and chronic SCI cohorts.	
		FES						
BeDell et al. [133] N = 12 M Phase 1: FES KE RT Phase 2: FES cycling progression Phase 3a: FES cycling Phase 3b: FES cycling + arm ergometry	C5-T12; AIS A; 9.7 ± 5.1 yr (3–19 yr)	Phase 1–3a: 34 ± 8 wk; 3 d/wk (actual 2.0 ± 0.3 d/wk); 30 min/d Phase 3b: 25 ± 9 wk; 3 d/wk; 30 min/d Training parameters: 10–132 mA, 30 Hz, 400 µs	Lumbar 2–4 aBMD Femur neck aBMD Ward’s triangle aBMD Femur trochanter aBMD	Baseline 1.27 g/cm^2^ 0.78 g/cm^2^ 0.71 g/cm^2^ 0.61 g/cm^2^	Phase 3a +1.6% +1.3% +0.0% +4.9%	Phase 3b +5.5% +5.1% −1.4% +0.0%		
Chen et al. [29] N = 15 M FES cycling, N = 15 M	C5-T8; AIS A; 2.6–15.7 yr	6 mo; 5 d/wk; 30 min/d Training parameters: 0–120 mA, 20 Hz, 300 µs	L2-L4 spine aBMD Femur neck aBMD Distal femur aBMD Proximal tibia aBMD Heel aBMD	Baseline 1.05 g/cm^2^ 0.69 g/cm^2^ 0.72 g/cm^2^ 0.55 g/cm^2^ 0.35 g/cm^2^	6 mo +0.1% −2.2% +11% * +13% * +3.2%	F/U 6 mo +0.1% −8.7% # −1.4% # −1.6% # −6.6% #		
Frotzler et al. [134] N = 10 M, 2 F FES RT + cycle	T3-T12; AIS A; 3.6–25.5 yr	12 mo (actual: 19 ± 2.1 mo); FES RT: 3–4 d/wk for 3 mo; 30–60 min/d; FES cycle: 5 d/wk for 9 mo; 60 min/d Training parameters: FES RT: 80–150 mA, 50 Hz, 300–400 µs, 5 s on/off FES cycles: 50 Hz, 0–500 µs	Femur Diaphysis cortical vBMD Distal epiphysis total vBMD Distal epiphysis trabecular vBMD Tibia Proximal total vBMD Proximal trabecular vBMD Diaphysis cortical vBMD Distal total vBMD Distal trabecular vBMD	Baseline 1102 mg/cm^3^ 158 mg/cm^3^ 122 mg/cm^3^ 124 mg/cm^3^ 71.6 mg/cm^3^ 1112 mg/cm^3^ 166 mg/cm^3^ 101 mg/cm^3^	6 mo −0.4% * +5.3% +9.3% −0.7% −2.8% +0.7% −0.7% −0.8%	12 mo −0.4% * +6.7% * +13% * −1.6% −3.4% +0.8% +0.3% +0.2%		
Griffin et al. [132] N = 13 M, 5 F FES cycling	C4-T7; Complete-Incomplete; 2–53 yr	10 wk; 2–3 d/wk; 30 min/d Training parameters: 0–140 mA, 50 Hz, 49 rpm	Total body bone mass	Baseline 6.03 lbs	10 wk −0.66%	Note: Data are bone mass (lbs), BMD not reported.		
Leeds et al. [135] N = 6 M FES KE RT + FES cycling	C4-C6; AIS A; 2–9 yr	1 mo RT; 3 d/wk; up to 45 KE then 6 mo cycle; 3 d/wk; up to 30 min/d Training parameters: 0–130 mA, ≤220 V, 30–60 Hz, 350 µs	Femur neck aBMD Ward’s triangle aBMD Trochanter aBMD	Baseline 0.65 g/cm^2^ 0.52 g/cm^2^ 0.46 g/cm^2^	6 mo −1.5% +0.0% −6.5%			
Mohr et al. [30] N = 8 M, 2 F FES cycle	C6-T4; Complete; 12.5 ± 2.7 yr (2–24 yr)	3 d/wk for 12 mo, then 1 d/wk for 6 mo; 30 min/d Training parameters: 0–130 mA, 30 Hz, 350 ms, 50 rpm	Femur neck aBMD Lumbar spine aBMD Proximal tibia aBMD	Baseline 0.63 g/cm^2^ 1.21 g/cm^2^ 0.49 g/cm^2^	12 mo −3.2% +0.8% +10% *	18 mo −13% +1.7% −2.0%		
Needham-Shropshire et al. [131] N = 13 M, 3 F FES + OGW	T4-T11; AIS A; >6 mo 3.8 yrs (avg)	20 wk; 3 d/wk; ≤120 min/d Training parameters: 0–300 mA, 24 Hz, 150 300 µs	G1: Femur neck aBMD G1: Ward’s triangle aBMD G1: Femur Trochanter aBMD	Baseline 0.77 g/cm^2^ 0.69 g/cm^2^ 0.58 g/cm^2^	12 wk −1.3% −1.5% −1.7%	20 wk −1.3% −1.5% 0%		
Rodgers et al. [108] N = 9 M, 3 F FES KE RT	C4-T10; incomplete and complete; 0.7–17 yr	12 wk/36 bouts (actual 12–18 wk); 3 d/wk (actual 2.5 d/wk); 30 min/d Training parameters: 100 mA, 35 Hz, 300 µs	Total cohort Distal tibia trabecular vBMD Chronic cohort Distal tibia trabecular vBMD	Baseline 235 mg/cm^3^ 214 mg/cm^3^	12–18 wk −5.4% −2.9%	Note: BMD determined on N = 1 <1 yr and N = 7 chronic SCI. Values are an average of total and chronic cohorts. Acute cohort data are in Table 2.		

BWSTT, bodyweight-supported treadmill training; RGO, reciprocating gait orthosis; FES, functional electrical stimulation; RT, resistance training; OGW, overground walking; F, female; M, male; C, cervical; T, thoracic; L, lumbar; AIS, American Spinal Injury Association Impairment Scale; SCI Duration: time since SCI in relation to intervention reported as range, mean ± SD, or mean and (range); aBMD, areal bone mineral density; vBMD, volumetric bone mineral density; avg, average; min, minute; h, hour; d, day; wk, week; mo, month; yr, year; N/R, not reported; F/U, follow-up after intervention complete; Note: % change was reported in individual papers or was manually calculated from data in tables and/or figures; * indicates a *p*-value of <0.05 vs. the baseline; # indicates <0.05 vs. the initial BMD assessment after the baseline; a lack of symbols indicates no statistical differences that were reported versus the baseline or between groups.

**Table 5:** Controlled interventional studies evaluating the effects of activity-based physical therapy (ABPT) and/or loading on bone mineral density (BMD) or T-scores in adults with chronic spinal cord injury (SCI).

Author; Citation; Sample Size/Sex; Group (G): Modality	SCI Level; Severity; Duration	Training Duration; Frequency; Time; Parameters	Skeletal Site Evaluated and Outcomes Reported	Baseline BMD/T-Score	BMD (% Difference) T-Score (Actual Change)		
		VIBRATION					
Dudley-Javoroski et al. [136] N = 4 M, 2 F G1: Unilateral seated vibration G2: Untrained limb	C7-T8; AIS A-B; 3.8–14.7 yr	12 mo; 3 d/wk; 20 min/d Training parameters: 0.6 g, 30 Hz, 10–15% body weight loading	G1: Distal femur (14–16%) vBMD G2: Distal femur (14–16%) vBMD G1: Distal femur (8–10%) vBMD G2: Distal femur (8–10%) vBMD G1: Distal femur (4–6%) vBMD G2: Distal femur (4–6%) vBMD G1: Distal tibia (4–6%) vBMD G2: Distal tibia (4–6%) vBMD G1: Distal tibia (8–10%) vBMD G2: Distal tibia (8–10%) vBMD G1: Distal tibia (14–16%) vBMD G2: Distal tibia (14–16%) vBMD	Baseline 43.9 mg/cm^3^ 70.8 mg/cm^3^ 45.5 mg/cm^3^ 37.0 mg/cm^3^ 63.7 mg/cm^3^ 54.4 mg/cm^3^ 124 mg/cm^3^ 105 mg/cm^3^ 106 mg/cm^3^ 105 mg/cm^3^ 95.9 mg/cm^3^ 116 mg/cm^3^	12 mo −28% −56% −5.2% −16% +2.9% −2.9% −18% −4.8% −24% −26% −13% −3.2%	Note: vBMD was assessed at multiple sites at the distal femur and proximal tibia. Skeletal sites are listed as the distance from the distal end of the femur or the proximal end of the tibia, as a % of whole bone length.	
Edwards et al. [101] N = 47 M, 14 F G1: Seated vibration, N = 14 M, 6 F G2: Teriparatide, M = 17 M, 3 F G3: Vibration + teriparatide, N = 16 M, 5 F	C-L; AIS A-D; 19 ± 13.8 yr	12 mo; Training frequency N/R; 10 min/d Training parameters: 0.5 g, 30 Hz	G1: Lumbar spine aBMD G2: Lumbar spine aBMD G3: Lumbar spine aBMD G1: Total hip aBMD G2: Total hip aBMD G3: Total hip aBMD G1: Femur neck aBMD G2: Femur neck aBMD G3: Femur neck aBMD G1: Proximal femur aBMD G2: Proximal femur aBMD G3: Proximal femur aBMD G1: Distal femur aBMD G2: Distal femur aBMD G3: Distal femur aBMD G1: Proximal tibia aBMD G2: Proximal tibia aBMD G3: Proximal tibia aBMD Femur (vBMD) G1: Epiphyseal trabecular vBMD G2: Epiphyseal trabecular vBMD G3: Epiphyseal trabecular vBMD G1: Metaphyseal trabecular vBMD G2: Metaphyseal trabecular vBMD G3: Metaphyseal trabecular vBMD Tibia (vBMD) G1: Epiphyseal trabecular vBMD G2: Epiphyseal trabecular vBMD G3: Epiphyseal trabecular vBMD G1: Metaphyseal trabecular vBMD G2: Metaphyseal trabecular vBMD G3: Metaphyseal trabecular vBMD	Baseline 1.00 g/cm^2^ 1.04 g/cm^2^ 1.02 g/cm^2^ 0.63 g/cm^2^ 0.66 g/cm^2^ 0.64 g/cm^2^ 0.63 g/cm^2^ 0.66 g/cm^2^ 0.62 g/cm^2^ 0.45 g/cm^2^ 0.50 g/cm^2^ 0.50 g/cm^2^ 0.62 g/cm^2^ 0.59 g/cm^2^ 0.61 g/cm^2^ 0.47 g/cm^2^ 0.44 g/cm^2^ 0.49 g/cm^2^ 70 mg/cm^3^ 50 mg/cm^3^ 50 mg/cm^3^ 20 mg/cm^3^ 00 mg/cm^3^ 00 mg/cm^3^ 50 mg/cm^3^ 30 mg/cm^3^ 30 mg/cm^3^ 20 mg/cm^3^ 00 mg/cm^3^ 00 mg/cm^3^	12 mo +1.7% +5.5% +4.8%* +0.6% +0.9% +0.4% −0.1% −0.6% +1.7% +2.8% +2.8% −0.5% −0.2% +0.5% −0.6% +2.7% +4.8% −0.4% +10% −24% −14% +16% −42% −29% * −68% −85% −33% −85% −30% −74%		
		BWSTT/OGW					
Mobarake et al. [97] N = 17 M G1: BWSTT + OGW + RT, N = 10 M G2: OGW + RT, N = 7 M	Level N/R; AIS B-C; >6 mo	12 wk; 4 d/wk; 60 min/d Training parameters: G1: 0–50% BWSTT, bodyweight support reduced over time, 0.3 km/h G2: OGW and RT	G1: Femur neck aBMD G2: Femur neck aBMD G1: Lumbar spine aBMD G2: Lumbar spine aBMD	Baseline 0.78 g/cm^2^ 0.75 g/cm^2^ 0.89 g/cm^2^ 0.85 g/cm^2^	12 wk +17% †† +1.3% +9.0% †† +1.2%		
		FES					
Belanger et al. [137] N = 22 M, 6 F G1: FES KE PRT limb, N = 11 M, 3 F G2: FES KE no PRT limb, N = 11 M, 3 F G3: Normative non-SCI, N = 11 M, 3 F	C5-T5; AIS A-C; 1.2–23 yr	24 wk; 5 d/wk; 1 h/d Training parameters: 10–150 mA, 25 Hz, 300 µs, 5 s on:off	G1: Distal femur aBMD G2: Distal femur aBMD G3: Distal femur aBMD G1: Proximal tibia aBMD G2: Proximal tibia aBMD G3: Proximal tibia aBMD G1: Tibia diaphysis aBMD G2: Tibia diaphysis aBMD G3: Tibia diaphysis aBMD	Baseline 0.7 g/cm^2^ 0.5 g/cm^2^ 0.4 g/cm^2^ 0.5 g/cm^2^ 0.4 g/cm^2^ 0.3 g/cm^2^ 1.5 g/cm^2^ 1.1 g/cm^2^ 1.1 g/cm^2^	24 wk +18% * +18% * N/R +15% * +15% * N/R +0.0% +0.0% N/R	Note: Post-training BMD change was not different between G1 and G2. % difference is an average change for G1 and G2 combined.	
Bloomfield et al. [96] N = 10 M, 7 F G1: FES KE RT + cycling, N = 5 M, 4 F G2: SCI controls, N = 5 M, 3 F	G1: C5-T7; Frankel A-B; 6 ± 1.2 yr G2: C4-T12; Frankel A-B; 8.3 ± 2.3 yr	5.9 ± 1.0 wk FES KE RT 6 mo FES cycling (80 sessions); 3 d/wk; 30 min/d Training parameters: 0–130 mA, 30 Hz, 350 ms, 50 rpm	G1: Lumbar spine aBMD G2: Lumbar spine aBMD G1: Femur neck aBMD G2: Femur neck aBMD G1: Distal femur aBMD G2: Distal femur aBMD G1: Proximal tibia aBMD G2: Proximal tibia aBMD	Baseline 1.23 g/cm^2^ 1.28 g/cm^2^ 0.80 g/cm^2^ 0.70 g/cm^2^ 0.47 g/cm^2^ 0.52 g/cm^2^ 0.36 g/cm^2^ 0.39 g/cm^2^	3 mo +1.6% +2.4% −2.1% −1.9% +6.8% +2.1% −2.8% −0.3%	6 mo +1.4% +0.4% −2.0% −1.7% +4.9% −2.3% −5.3% −3.9%	9 mo +3.8% * N/R −3.9% N/R +10% N/R +4.2% N/R
Craven et al. [98] N = 34 M G1: BWSTT + FES, N = 14 M, 3 F G2: Aerobic + RT, N = 12 M, 5 F	C2-T12; AIS C-D; ≥18 mo G1: 5 ± 6.6 yr G2: 5 ± 18 yr	4 mo; 3 d/wk; 45 min/d Training parameters: G1: 8–125 mA, 20–50 Hz, 0–250−300 µs BWSTT individualized bodyweight support reduced and speed increased as training progressed G2: 20–25 min aerobic exercise (arm or leg cycling, walking in parallel bars or on treadmill), 2–3 sets of 12–15 reps RT for muscles capable of voluntary contraction	G1: Total hip aBMD G2: Total hip aBMD G1: Distal femur aBMD G2: Distal femur aBMD G1: Proximal tibia aBMD G2: Proximal tibia aBMD G1: Tibial diaphysis cortical vBMD G2: Tibial diaphysis cortical vBMD G1: Tibial diaphysis trabecular vBMD G2: Tibial diaphysis trabecular vBMD G1: Distal tibia trabecular vBMD G2: Distal tibia trabecular vBMD	Baseline 0.89 g/cm^2^ 0.86 g/cm^2^ 0.89 g/cm^2^ 0.81 g/cm^2^ 0.71 g/cm^2^ 0.68 g/cm^2^ 1089 g/cm^3^ 1107 g/cm^3^ 87.7 g/cm^3^ 88.5 g/cm^3^ 202 g/cm^3^ 171 g/cm^3^	4 mo −2.2% +1.2% −2.2% −2.4% +0.0% −4.3% −0.1% +0.5% +4.0% −0.3% −6.4% −3.5%	F/U 12 mo −1.1% +4.7% −2.2% +0.0% −2.8% −1.4% −0.6% +0.5% +1.7% +6.5% −0.5% +4.6%	
Hangartner et al. [140] N = 30 M, 7 F (sex N/R for groups) G1: FES KE RT or FES cycling, N = 15 G2: SCI control, N = 22	C5-T10; Severity N/R; 0.3–15.4 yr	12 wk cycles [n = 9 underwent 1, 2 (n = 3), 3 (n = 2), 4 (n = 1) additional 12 wk blocks]; 3 d/wk; 30 min/d Training parameters: FES KE: 25% max weight, 2 sets x 30 reps, 5 min rest, then 12.5% max weight, 60 reps or to fatigue FES Cycle: 80–130 mA, 35 Hz, 375 µs, 50 rpm, 0–36.4 W power output	G1: Proximal tibia cortical vBMD G2: Proximal tibia cortical vBMD G1: Proximal tibia sub-cortical vBMD G2: Proximal tibia sub-cortical vBMD G1: Proximal tibia trabecular vBMD G2: Proximal tibia trabecular vBMD G1: Distal tibia cortical vBMD G2: Distal tibia cortical vBMD G1: Distal tibia sub-cortical vBMD G2: Distal tibia sub-cortical vBMD G1: Distal tibia trabecular vBMD G2: Distal tibia trabecular vBMD	Baseline N/R N/R N/R N/R N/R N/R N/R N/R N/R N/R N/R N/R	Per Yr +0.2% +1.0% +3.4% † +1.7% † +2.8% †† +2.7% ††	Note: % difference represents the estimated % less bone loss occurring per year in G1 vs. G2. For example, 0.2% indicates that G1 exhibited an estimated 0.2% less bone loss per yr when compared with G2. Study included N = 2 <1 yr and N = 13 chronic SCI. Values not available for acute/subacute vs. chronic.	
Johnston et al. [139] N = 14 M, 3 F (sex N/R for groups) G1: FES cycling high cadence, N = 8 G2: FES cycling low cadence, N = 9	C4-T6; AIS A-B; 12 ± 10 yr (1–27.5 yr)	6 mo; 3 d/wk; 1 h/d Training parameters: FES: 0–140 mA, 33 Hz, 250 µs against increasing resistance G1: 50 rpm G2: 20 rpm	G1: Distal femur aBMD G2: Distal femur aBMD	Baseline 0.80 g/cm^2^ 0.67 g/cm^2^	6 mo −13% −6.0%		
Morse et al. [141] N = 18M, 2F (sex N/R for groups) G1: FES RT + rowing + ZA, N = 10 G2: FES RT + FES rowing, N = 10	C4 or lower; AIS A-C; 11.6 ± 12.7 yr (0.4–37.9 yr)	FES RT: 2–12 wk; 3–5 d/wk FES ROW: 12 mo; 3 d/wk (actual 1.6 ± 0.1 d/wk); 30 min/d Training parameters: FES RT: 0–110 mA, 40 Hz, 450 ms, 6 s per contraction Row: 40 Hz, 450 ms	G1: Total hip aBMD G2: Total hip aBMD G1: Femur neck aBMD G2: Femur neck aBMD G1: Distal femur aBMD G2: Distal femur aBMD G1: Proximal tibia aBMD G2: Proximal tibia aBMD	Baseline 0.77 g/cm^2^ 0.82 g/cm^2^ 0.82 g/cm^2^ 0.85 g/cm^2^ 0.76 g/cm^2^ 0.83 g/cm^2^ 0.76 g/cm^2^ 0.82 g/cm^2^	12 mo N/R N/R N/R N/R N/R N/R N/R N/R	Note: BMD determined on N = 2 <1 yr and N = 18 chronic SCI. Values are the average of the total cohort. Data on acute/subacute versus chronic SCI was not reported.	
Shields et al. [138] N = 4 M G1: FES RT trained limb G2: Untrained limb	T1-T7; AIS A; 2–12 yr	6–11 mo; 5 d/wk; 30 min/d Training parameters: 0–200 mA, 400 V, 10-pulse train (15 Hz, 667 ms) every 2 s, 125 total trains, 89–116% bodyweight compressive load	G1: Proximal tibia aBMD G2: Proximal tibia aBMD	Baseline 0.20 g/cm^2^ 0.21 g/cm^2^	6–11 mo +3.1% −6.9%		

G, group; BWSTT, bodyweight-supported treadmill training; FES, functional electrical stimulation; RT, resistance training; PRT, progressive resistance training; OGW, overground walking; ZA, zoledronic acid; F, female; M, male; C, cervical; T, thoracic; L, lumbar; AIS, American Spinal Injury Association Impairment Scale; SCI Duration: time since SCI in relation to intervention reported as range, mean ± SD, or mean and (range); aBMD, areal bone mineral density; vBMD, volumetric bone mineral density; min, minute; h, hour; d, day; wk, week; mo, month; yr, year; N/R, not reported; Note: % change was reported in individual papers or was manually calculated from data in tables and/or figures; * indicates a *p*-value of <0.05 vs. the baseline; † indicates <0.05, †† <0.01 between the groups; a lack of symbols indicates no statistical differences were reported versus the baseline or between the groups.

**Table 6:** Interventional studies evaluating the effects of activity-based physical therapy (ABPT) and/or loading on bone microstructural in adults with subacute and/or chronic spinal cord injury (SCI).

Author; Citation; Sample Size/Sex; Group (G): Modality	SCI Level; Severity; Duration	Training Duration; Frequency; Time; Parameters	Skeletal Site Evaluated and Outcomes Reported	Baseline Values	% Difference		
		FES					
Craven et al. [98] N = 34 M G1: BWSTT + FES, N = 14 M, 3 F G2: Aerobic training + RT, N = 12 M, 5F	C2-T12; AIS C-D; ≥18 mo G1: 5 ± 6.6 yr G2: 5 ± 18 yr	4 mo; 3 d/wk; 45 min/d Training parameters: G1: 8–125 mA, 20–50 Hz, 0–250−300 µs BWSTT individualized bodyweight support reduced and speed increased as training progressed G2: 20–25 min aerobic exercise (arm or leg cycling, walking in parallel bars or on treadmill), 2–3 sets of 12–15 reps RT for muscles capable of voluntary contraction	Tibial Diaphysis G1: Cortical thickness G2: Cortical thickness	Baseline 3.88 mm 4.14 mm	4 mo −1.3% +1.7%	F/U 12 mo −5.2% −1.4%	
Johnston et al. [139] N = 14 M, 3 F (sex N/R for groups) G1: FES cycling high cadence, N = 8 G2: FES cycling low cadence, N = 9	C4-T6; AIS A-B; 12 ± 10 yr (1–27.5 yr)	6 mo; 3 d/wk; 1 h/d Training parameters: FES: 0–140 mA, 33 Hz, 250 µs against increasing resistance G1: 50 rpm G2: 20 rpm	Distal Femur G1: Trabecular bone volume G2: Trabecular bone volume G1: Trabecular number G2: Trabecular number G1: Trabecular thickness G1:Trabecular thickness G1: Trabecular spacing G2: Trabecular spacing G1: Cortical bone volume G2: Cortical bone volume	Baseline 19.2% 21.1% 1.17/mm 1.08/mm 0.179 mm 0.171 mm 0.71 mm 0.85 mm 50.7 mm^3^ 50.5 mm^3^	6 mo −2.8% +3.3% −2.6% +4.6% −1.1% +0.0% +2.8% −9.4% −3.4% −0.8%		
Morse et al. [141] N = 18 M, 2 F (sex N/R for groups) G1: FES RT + rowing + ZA, N = 10 G2: FES RT + rowing, N = 10	C4 or lower; AIS A-C; 11.6 ± 12.7 yr (0.4–37.9 yr)	FES RT: 2–12 wk; 3–5 d/wk FES Row: 12 mo; 3 d/wk (actual 1.6 ± 0.1 d/wk); 30 min/d Training parameters: FES RT: 0–110 mA, 40 Hz, 450 ms, 6 s per contraction FES row: no ramp, 40 Hz, 450 ms	Distal Femur G1: Cortical bone volume G2: Cortical bone volume G1: Cortical thickness G2: Cortical thickness Proximal Tibia G1: Cortical bone volume G2: Cortical bone volume G1: Cortical thickness G2: Cortical thickness	Baseline 12.5 cm^3^ 11.9 cm^3^ 0.3 mm 0.3 mm 13.2 cm^3^ 13.3 cm^3^ 0.13 mm 0.14 mm	12 mo +1.7% † +1.4% +0.3% −1.0% +0.1% †† −5.7% +0.0% −7.0%		
		STANDING/VIBRATION					
Dudley-Javoroski et al. [136] N = 4 M, 2 F G1: Unilateral seated vibration G2: Untrained limb	C7-T8; AIS A-B; 3.75–14.7 yr	12 mo; 3 d/wk; 20 min/d Training parameters: 0.6 g, 30 Hz, 10–15% body weight loading	Distal Femur (14–16% length) G1: Trabecular thickness G2: Trabecular thickness Distal Femur (8–10% length) G1: Trabecular thickness G2: Trabecular thickness Distal Femur (4–6% length) G1: Trabecular thickness G2: Trabecular thickness Distal Tibia (4–6% length) G1: Trabecular thickness G2: Trabecular thickness Distal Tibia (8–10% length) G1: Trabecular thickness G2: Trabecular thickness Distal Tibia (14–16% length) G1: Trabecular thickness G2: Trabecular thickness	Baseline 0.205 mm 0.241 mm 0.101 mm 0.088 mm 0.133 mm 0.124 mm 0.126 mm 0.129 mm 0.129 mm 0.127 mm 0.547 mm 0.341 mm	12 mo −52% −56% +20% +23% −14% −7.7% −2.1% −5.8% +1.1% +8.9% −1.2% +41%		
Edwards et al. [101] N = 47 M, 14 F G1: Seated vibration, N = 14 M, 6 F G2: TA, M = 17 M, 3 F G3: Seated vibration + TA, N = 16 M, 5 F	C-L; AIS A-D; 19 ± 13.8 yr	12 mo; Frequency N/R; 10 min/d Training parameters: 0.5 g, 30 Hz	Femur Diaphysis G1: Cortical bone volume G2: Cortical bone volume G3: Cortical bone volume Distal Femur Metaphysis G1: Cortical bone volume G2: Cortical bone volume G3: Cortical bone volume Distal Femur Epiphysis G1: Cortical bone volume G2: Cortical bone volume G3: Cortical bone volume Proximal Tibia Epiphysis G1: Cortical bone volume G2: Cortical bone volume G3: Cortical bone volume Proximal Tibia Metaphysis G1: Cortical bone volume G2: Cortical bone volume G3: Cortical bone volume Tibia Diaphysis G1: Cortical bone volume G2: Cortical bone volume G3: Cortical bone volume	Baseline 13.0 cm^3^ 13.9 cm^3^ 13.2 cm^3^ 10.4 cm^3^ 11.5 cm^3^ 11.3 cm^3^ 2.95 cm^3^ 2.43 cm^3^ 2.52 cm^3^ 2.64 cm^3^ 2.79 cm^3^ 3.17 cm^3^ 11.8 cm^3^ 11.9 cm^3^ 11.9 cm^3^ 13.2 cm^3^ 14.7 cm^3^ 14.0 cm^3^	12 mo −0.1% +0.2% −0.1% +1.1% +3.8% +1.7% +3.8% +11% +1.3% +10% +14% +7.1% +0.9% +1.7% +2.1% +22% +0.8% +0.4%		
Wuermser et al. [102] N = 5 M, 4 F Standing + vibration	AIS A-B; T3-T12; 2–27 yr	6 mo; 5 d/wk; 20 min/d Training parameters: 0.3 g, 34 Hz sinusoidal movement of 50 µm w/lower extremities supporting ~86% body weight	Distal Tibia Trabecular number Trabecular thickness Trabecular separation Cortical thickness Cortical area	Baseline 1.09/mm 0.04 mm 1.15 mm 0.80 mm 86.5 mm^2^	3 mo +0.9% +0.0% +8.7% −2.5% −3.3%	6 mo −5.5% +0.0% +7.6% −1.3% −1.8%	F/U 6 mo −7.3% +0.0% +15% −3.8% −3.5%

G, group; BWSTT, bodyweight supported treadmill training; FES, functional electrical stimulation; RT, resistance training; ZA, zoledronic acid; TA, teriparatide F, female; M, male; C, cervical; T, thoracic; L, lumbar; AIS, American Spinal Injury Association Impairment Scale; SCI Duration: time since SCI in relation to intervention reported as range, mean ± SD, or mean and (range); avg, average; min, minute; h, hour; d, day; wk, week; mo, month; yr, year; N/R, not reported; FEA, finite element analysis; F/U, follow-up after intervention complete; Note: % change was reported in individual papers or was manually calculated from data in tables and/or figures; † indicates <0.05, †† <0.01 between groups; a lack of statistical symbols indicates no statistical differences that were reported versus the baseline or between the groups.

## Data Availability

The data presented in this study are available on request from the corresponding author. The data are not publicly available due to data being property of the United States federal government.
